# Supplementary material for: The effects of temperature on nestling growth in a songbird depend on developmental constraints
Source: PLoS One. 2026 Apr 22;21(4):e0334815. doi: 10.1371/journal.pone.0334815 (PMC13102239; doi:10.1371/journal.pone.0334815)
Supplement: S1 Fig — A boxplot of feeding BLUPs, measured in visits per hour, is provided for each of two levels of parental care (‘low,’ ‘high’) used for stratified analyses in question three. (PDF) [file pone.0334815.s001.pdf]

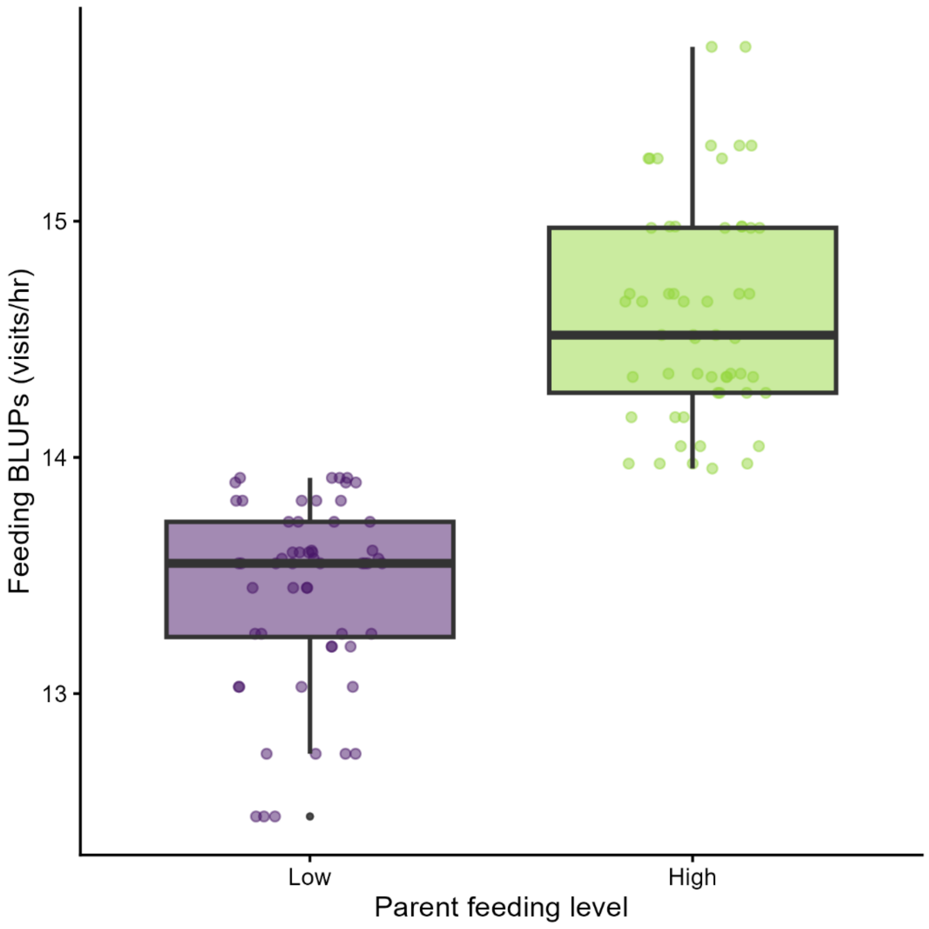

S1 Fig. Best unbiased linear predictions (BLUPs) for the feeding rate (visits/hour) at each nest across development in wild barn swallows. A boxplot of feeding BLUPs, measured in visits per hour, is provided for each of two levels of parental care (‘low,’ ‘high’) used for stratified analyses in question three.
