# Supplementary material for: The effects of temperature on nestling growth in a songbird depend on developmental constraints
Source: PLoS One. 2026 Apr 22;21(4):e0334815. doi: 10.1371/journal.pone.0334815 (PMC13102239; doi:10.1371/journal.pone.0334815)
Supplement: S2 Fig — Daily minimum temperature (A), maximum temperature (B), and temperature variability (interquartile range) (C) in °Celsius recorded by Govee thermometers near each barn swallow nest during the nestling rearing period. Each color corresponds to one of seven breeding sites. Lines connect daily measures for each individual nest. (PDF) [file pone.0334815.s002.pdf]

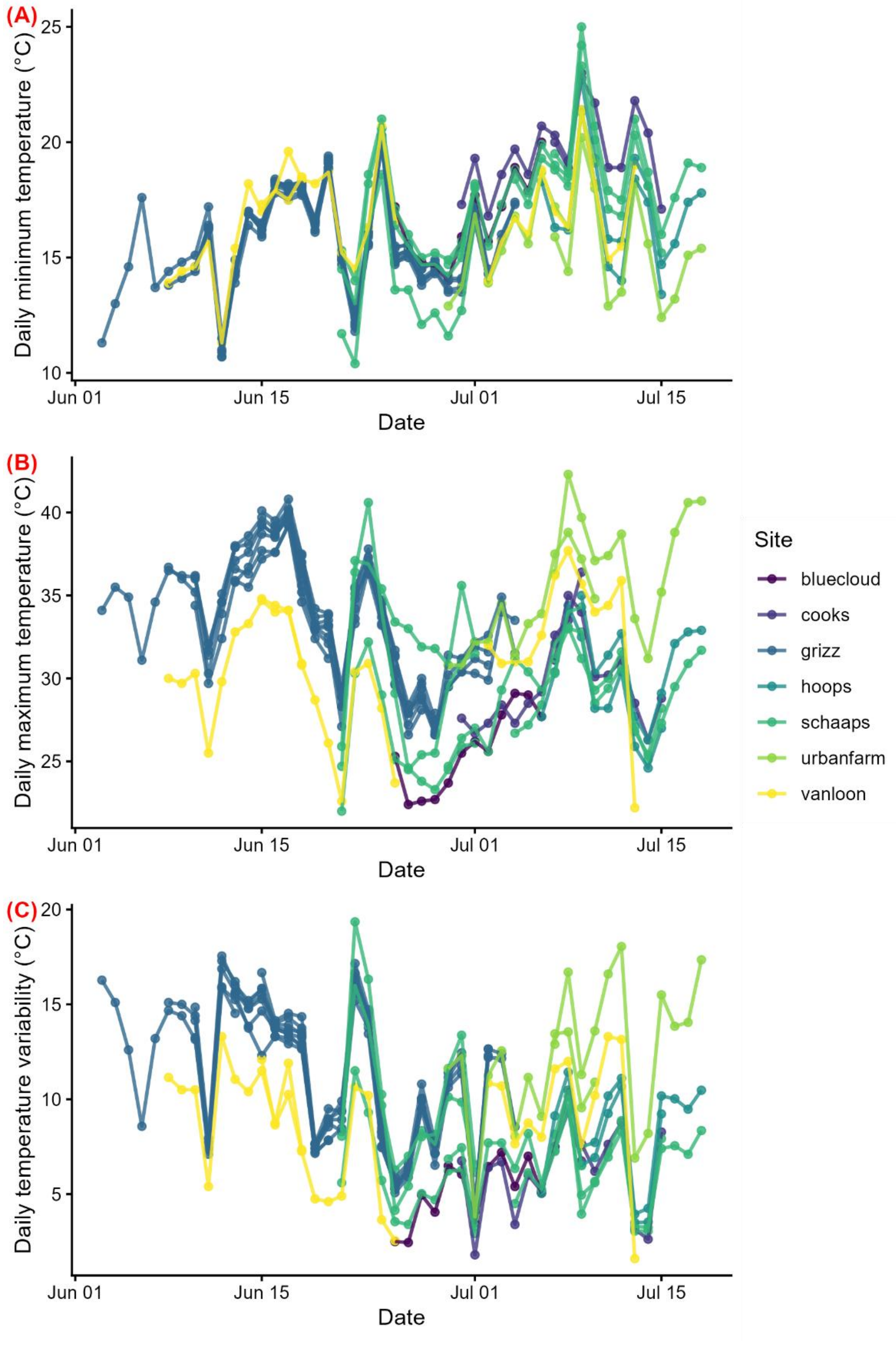

S2 Fig. Daily temperatures recorded at wild barn swallow nests in Boulder County, CO. Daily minimum temperature (**A**), maximum temperature (**B**), and temperature variability (interquartile range) (**C**) in °C recorded by Govee thermometers near each barn swallow nest during the nestling rearing period. Each color corresponds to one of seven breeding sites. Lines connect daily measures for each individual nest.
