# Supplementary material for: The effects of temperature on nestling growth in a songbird depend on developmental constraints
Source: PLoS One. 2026 Apr 22;21(4):e0334815. doi: 10.1371/journal.pone.0334815 (PMC13102239; doi:10.1371/journal.pone.0334815)
Supplement: S3 Fig — Predicted relationships of minimum temperature (A), maximum temperature (B), and temperature variability (interquartile range) (C) across the nestling period and nestling mass from linear mixed models, stratified by three levels of parent feeding—low (‘Low’, n = 35), medium (‘Med’, n = 36), and high (‘High’, n = 35). Model predictions are displayed as lines, and raw data are displayed as points. Colors, line types, and shapes correspond to the level of parent feeding. In unstratified analyses (not visualized), there was evidence for an interaction between minimum temperature and parental feeding (βmed = 1.04 g per 1 SD ℃ [95% CI: −0.59, 2.53], p = 0.19; βhigh = −1.09 g per 1 SD ℃ [95% CI: −2.49, 0.28], p = 0.14; F-test for overall effect: p = 0.03). There was no evidence for an interaction between maximum temperature and parental feeding level (βmed = −0.63 g per 1 SD ℃ [95% CI: −1.98, 0.66], p = 0.36; βhigh = 0.80 g per 1 SD ℃ [95% CI: −0.87, 2.48, p = 0.36; F-test for overall effect: p = 0.24) or between temperature variability and parental feeding level (βmed = −0.80 g per 1 SD ℃ [95% CI: −2.02, 0.42, p = 0.20; βhigh = 0.84 g per 1 SD ℃ [95% CI: −0.85, 2.55], p = 0.33; F-test for overall effect: p = 0.15). (PDF) [file pone.0334815.s003.pdf]

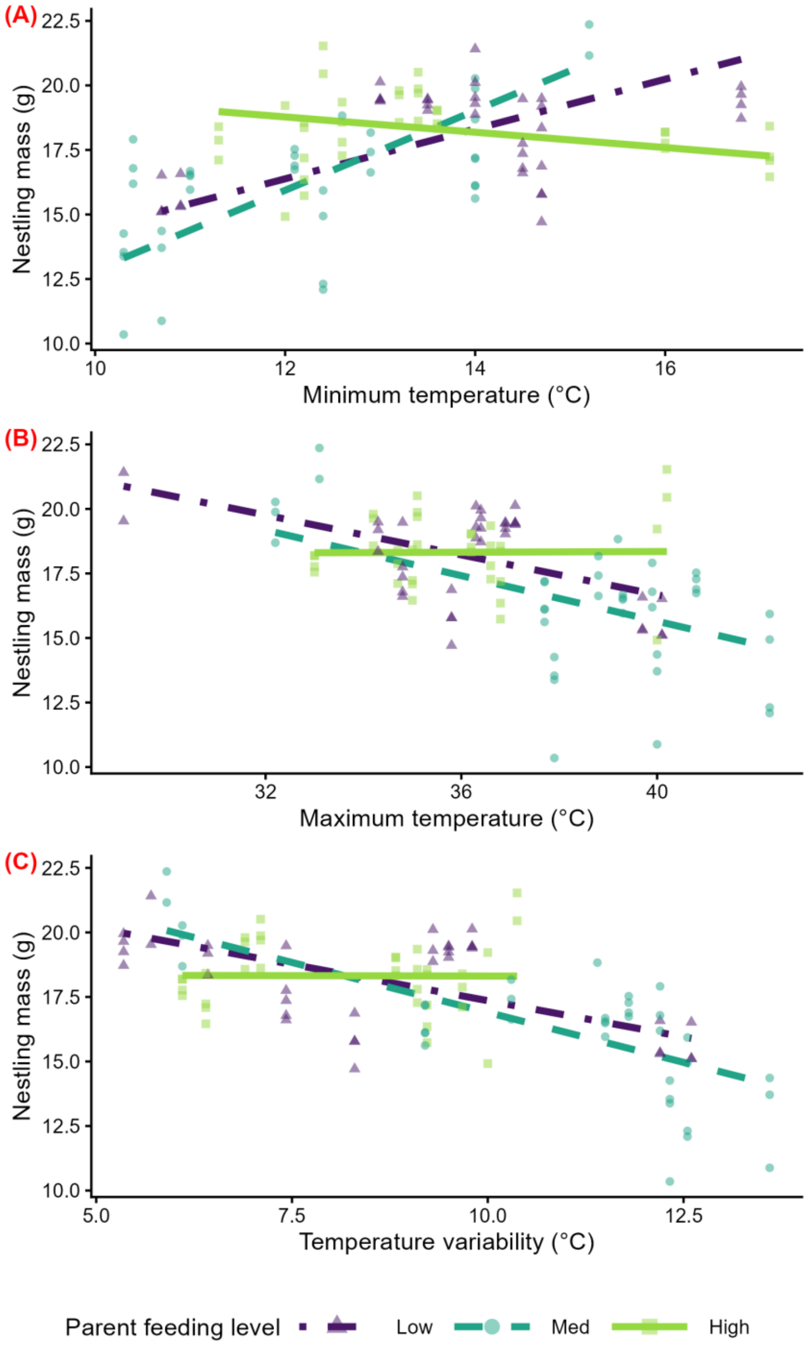

S3 Fig. Temperature effects on the mass of 11–13-day-old nestlings at nests receiving low, medium, or high parental feeding. Predicted relationships of minimum temperature (A), maximum temperature (B), and temperature variability (interquartile range) (C) across the nestling period and nestling mass from linear mixed models, stratified by three levels of parent feeding—low (‘Low’, n = 35), medium (‘Med’, n = 36), and high (‘High’, n = 35). Model predictions are displayed as lines, and raw data are displayed as points. Colors, line types, and shapes correspond to the level of parent feeding. In unstratified analyses (not visualized), there was evidence for an interaction between minimum temperature and parental feeding (β_{med} =

1.04 g per 1 SD ℃ [95% CI: -0.59, 2.53], $p = 0.19$; β_{high} = -1.09 g per 1 SD ℃ [95% CI: -2.49, 0.28], $p = 0.14$; F-test for overall effect: $p = 0.03$). There was no evidence for an interaction between maximum temperature and parental feeding level (β_{med} = -0.63 g per 1 SD ℃ [95% CI: -1.98, 0.66], $p = 0.36$; β_{high} = 0.80 g per 1 SD ℃ [95% CI: -0.87, 2.48, $p = 0.36$; F-test for overall effect: $p = 0.24$) or between temperature variability and parental feeding level (β_{med} = -0.80 g per 1 SD ℃ [95% CI: -2.02, 0.42, $p = 0.20$; β_{high} = 0.84 g per 1 SD ℃ [95% CI: -0.85, 2.55], $p = 0.33$; F-test for overall effect: $p = 0.15$).
