# Supplementary material for: The effects of temperature on nestling growth in a songbird depend on developmental constraints
Source: PLoS One. 2026 Apr 22;21(4):e0334815. doi: 10.1371/journal.pone.0334815 (PMC13102239; doi:10.1371/journal.pone.0334815)
Supplement: S4 Fig — This photo, taken during the study, is representative of the features of the nests and nest sites of the birds we monitored. (PDF) [file pone.0334815.s004.pdf]

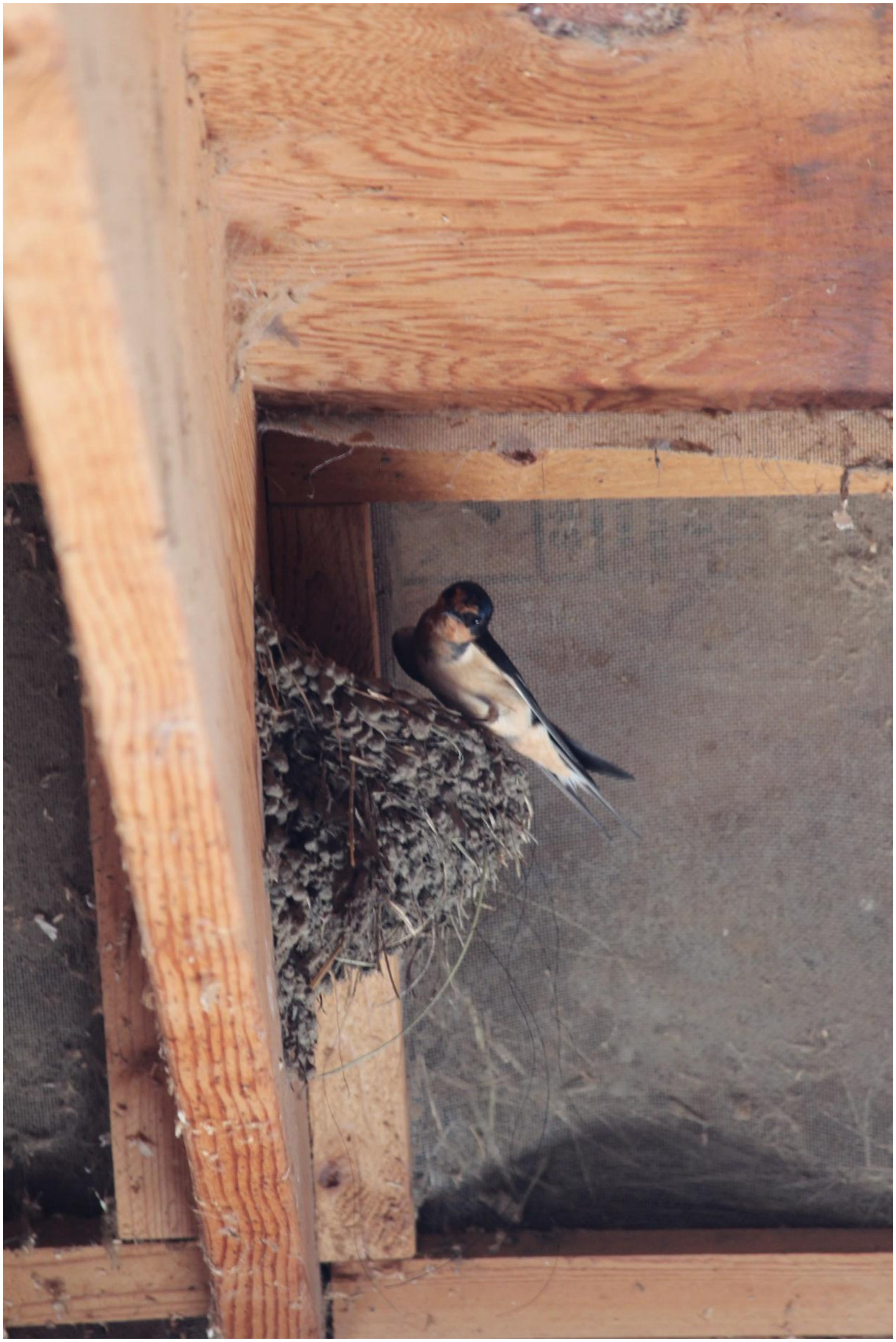

S4 Fig. Photo of a barn swallow perched on a mud cup nest. This photo, taken during the study, is representative of the features of the nests and nest sites of the birds we monitored.
