# Supplementary material for: The effects of temperature on nestling growth in a songbird depend on developmental constraints
Source: PLoS One. 2026 Apr 22;21(4):e0334815. doi: 10.1371/journal.pone.0334815 (PMC13102239; doi:10.1371/journal.pone.0334815)
Supplement: S1 Table — Each documented behavior has a corresponding location, type (state versus event), description, and developmental context. This ethogram was used for parental care observations. (PDF) [file pone.0334815.s005.pdf]

S1 Table. Ethogram of barn swallow parental care behaviors. Each documented behavior has a corresponding location, type (state versus event), description, and developmental context. This ethogram was used for parental care observations.

Behavior location	Behavior name	Behavior type	Description	Developmental context
At nest	Feeding	Event	The parent delivers food as indicated by putting its beak into the mouth of the nestling. Prey often cannot be seen in the parent's bill. Feeding usually occurs immediately after arrival at the nest.	Day 3: nestlings are small, so they usually cannot be seen by the observer. Feeding visits are identified by the parent leaning into the nest immediately after arrival from foraging
				Day 8: the parent can often be seen inserting its bill into the gap of nestlings. The parent usually still leans into the nest slightly when feeding.
				Day 12: nestlings can be seen, and feeding occurs without leaning into the nest.
	Brooding	State	The parent settles itself all the way into the nest on top of the nestlings. The parent may turn around in the nest while brooding. Brooding behavior looks similar across nestling ages but is far more common with younger nestlings (Day 3).	NA
	Sanitizing	State	The parent removes nestling fecal sacs or rearranges nestlings and feathers in the nest. The parent may carry fecal sacs out of the nest or consume them. When rearranging	Day 3: The parents often sanitize while settled in the nest cup. The parent will shift around, and the tail will twitch. The head will duck down into the nest.

			feathers or nestlings, the parent may perch on the edge of the nest and lean in deeply, tail twitching (see-saw motion; head and tail pivot up and down). The parent may also sanitize while brooding, as indicated by their head in the nest and tail twitching.	Days 8 and 12: sanitizing more commonly occurs while perched on the nest edge. Parents may be seen carrying fecal sacks away.
	Preening	State	The parent uses the beak to clean, stroke, or re-position its feathers while perched on the nest rim or settled in the nest, brooding.	NA
	Perching	State	The parent stands with feet on the side of the nest, not engaged in any other behaviors. The parent may look around the barn, or it may peer inside the nest.	NA
Away from nest	Near nest	State	The parent is visible at their typical perch location(s). Importantly, here it is reasonable to assume that the bird can see the nest (i.e., they are able to be vigilant of any nest threats).	NA
	Far from nest	State	The parent flies away from the nest and is either observed leaving the barn or heading towards a barn opening, but that might be out of sight for the observer.	NA
	Unknown	State	It is not known if the parent can reasonably see the nest or is in the barn at all.	NA
