# Supplementary material for: The effects of temperature on nestling growth in a songbird depend on developmental constraints
Source: PLoS One. 2026 Apr 22;21(4):e0334815. doi: 10.1371/journal.pone.0334815 (PMC13102239; doi:10.1371/journal.pone.0334815)
Supplement: S2 Table — Total number of feeding visits, nestling mass, and nestling wing length are separately estimated at three time points (days 3–4, 8–9, and 11–13). Temperature variability is defined as the interquartile range. The table provides the sample size for each variable at the level at which it was collected (n), as well as the mean, standard deviation (SD), minimum, and maximum. (PDF) [file pone.0334815.s006.pdf]

Supplemental Table 2. Background characteristics of wild nestling barn swallows in Boulder County, CO. Total number of feeding visits, nestling mass, and nestling wing length are separately estimated at three time points (days 3-4, 8-9, and 11-13). Temperature variability is defined as the interquartile range. The table provides the sample size for each variable at the level at which it was collected (n), as well as the mean, standard deviation (SD), minimum, and maximum.

Variable	n	Mean	SD	Minimum	Maximum
**Near-nest temperature (°C)**					
Mean temperature	31	23.99	1.32	21.17	26.05
Minimum temperature	31	13.10	1.80	10.30	17.10
Maximum temperature	31	36.89	2.96	29.10	42.30
Temperature variability	31	9.26	2.43	5.35	13.60
**Other nest characteristics**					
Brood size (number of nestlings)	31	3.42	0.96	1.00	5.00
Hatch date (days since June 1)	31	20.58	10.05	2.00	38.00
Nest age at last measure (days since hatch)	31	12.26	0.51	11.00	13.00
**Total feeding rate (counts/hour) across development**[1]					
Days 3-4	30	12.21	6.92	3.00	35.73
Days 8-9	28	12.73	8.08	2.97	29.92
Days 11-13	30	21.62	12.94	3.98	60.35
**Right wing length (mm) across development**					
Days 3-4	113	9.40	1.94	5.00	13.50
Days 8-9	108	30.71	5.36	15.00	40.50
Days 11-13	106	52.78	6.44	31.00	67.00
**Nestling mass (g) across development**					
Days 3-4	113	4.00	1.30	1.25	7.73
Days 8-9	108	12.95	2.56	6.01	17.66
Days 11-13	106	17.57	2.21	10.35	22.36

[1]Parental feeding behaviors are measured at the level of the nest and include the totals for both social parents (maternal and paternal care).
