# Supplementary material for: The effects of temperature on nestling growth in a songbird depend on developmental constraints
Source: PLoS One. 2026 Apr 22;21(4):e0334815. doi: 10.1371/journal.pone.0334815 (PMC13102239; doi:10.1371/journal.pone.0334815)
Supplement: S3 Table — Temperature variability is defined as the interquartile range. For each temperature variable, results are provided for unadjusted and adjusted models. Sample size for each developmental stage is provided in the header (n). For each model, the table provides the effect size and 95% confidence interval for temperature effects (β (95% CI)), and the corresponding degrees of freedom, t-value, and p-value from a two-tailed t-test using a Satterthwaite degree of freedom estimation. (PDF) [file pone.0334815.s007.pdf]

S3 Table. Associations of three temperature variables in early and late development (before or after six days post-hatch) with nestling mass. Temperature variability is defined as the interquartile range. For each temperature variable, results are provided for unadjusted and adjusted models. Sample size for each developmental stage is provided in the header (n). For each model, the table provides the effect size, standard error, and 95% confidence interval for temperature effects (β ± SE (95% CI)), and the corresponding degrees of freedom, t-value, and p-value from a two-tailed t-test using a Satterthwaite degree of freedom estimation.

	Early development models (n = 106)				Late development models (n = 106)			
Type	β ± SE (95% CI)[1]	DF	t-value	p-value	β ± SE (95% CI)[1]	DF	t-value	p-value
**Effect of minimum temperature (g per 1 SD °C)**								
Unadjusted	1.11 ± 0.35 (0.45, 1.80)	28.58	3.22	0.003	0.50 ± 0.38 (-0.22, 1.25)	28.55	1.30	0.20
Adjusted[2]	1.16 ± 0.42 (0.32, 2.00)	26.96	2.79	0.01	0.29 ± 0.38 (-0.45, 1.06)	26.96	0.75	0.46
**Effect of maximum temperature (g per 1 SD °C)**								
Unadjusted	-0.87 ± 0.33 (-1.53, -0.24)	29.13	-2.61	0.01	-1.16 ± 0.33 (-1.82, -0.53)	28.51	-3.56	0.001
Adjusted[3]	-0.66 ± 0.33 (-1.32, -0.04)	27.67	-2.03	0.05	-1.13 ± 0.31 (-1.76, -0.52)	26.87	-3.60	0.001
**Effect of temperature variability (g per 1 SD °C)**								
Unadjusted	-1.32 ± 0.29 (-1.9, -0.78)	28.61	-4.53	0.0001	-1.39 ± 0.29 (-1.99, -0.85)	28.45	-4.74	0.0001
Adjusted[4]	-1.41 ± 0.33 (-2.07, -0.77)	27.00	-4.21	0.0003	-1.33 ± 0.32 (-1.95, -0.70)	26.38	-4.14	0.0003

[1]Estimated β ± SE (95% CI) from linear mixed models in which temperature in early or late development are the explanatory variables of interest, nestling mass is the outcome of interest, and nest ID was included as a random intercept. Adjusted models include hatch date and number of nestlings in the nest. Continuous predictors are z-score standardized.

[2]R-squared for adjusted minimum temperature models. Early model: Marginal R-squared = 0.32, Conditional R-squared = 0.82; Late model: Marginal R-squared = 0.20, Conditional R-squared = 0.83

[3]R-squared for adjusted maximum temperature models. Early model: Marginal R-squared = 0.26, Conditional R-squared = 0.82; Late model: Marginal R-squared = 0.38, Conditional R-squared = 0.82

[4]R-squared for adjusted temperature variability models. Early model: Marginal R-squared = 0.42, Conditional R-squared = 0.81; Late model: Marginal R-squared = 0.42, Conditional R-squared = 0.81
