# Supplementary material for: The effects of temperature on nestling growth in a songbird depend on developmental constraints
Source: PLoS One. 2026 Apr 22;21(4):e0334815. doi: 10.1371/journal.pone.0334815 (PMC13102239; doi:10.1371/journal.pone.0334815)
Supplement: Attachment — Submitted filename: nestling_temperature_ms_pt_by_pt_final.docx [file pone.0334815.s012.docx]

**Point-by-point response to the editor’s and reviewers’ comments**

**Editor**

**Comment.** Thank you for submitting your manuscript to PLOS ONE. After careful consideration, we feel that it has merit but does not fully meet PLOS ONE’s publication criteria as it currently stands. Therefore, we invite you to submit a revised version of the manuscript that addresses the points raised during the review process.

While both reviewers agree that this study addresses an important knowledge gap, reviewer 1 requested more details on methodology. In particular, please: provide the rationale for comparison between the smallest nestlings and others (instead of dividing nestlings into small-sized and large-sized groups); and describe correlations and interactions between variables if any. Additionally, it would be great if you could address their query on minor technical points.

**Response.** Thank you for your comments. Below and in the revised text, we address these suggestions.

**Reviewer #1**

**Comment.** Overall: The current study focuses on air temperature on nestling body mass in the barn swallow. Authors studied whether the effect of temperature depends on localized early-life developmental constraints imposed by the timing of thermoregulatory development, competition with brood mates, and the amount of parental care, which has received limited attentions. However, the current manuscript suffers at least two issues. The first issue is the lack of explanation. For example, I could not understand why authors compared between the smallest nestlings and others (instead of dividing nestlings into small-sized and large-sized groups). I guess, there must be a good reason to use this classification, but authors should carefully explain the logic. Likewise, I could not understand why authors did not show mean temperature. And I could not understand why authors categorized high/med/low parental care instead of high/low parental care. Authors should also carefully explain correlations between variables. When correlation between variables is high, their relationships with dependent variables might not be independent (and thus they should be analyzed together, rather than separately). Second, authors should analyze interactions between variables. Statistical significance depends on sample size, and thus significant relationship in some groups but not in others does not always mean they differ in slope (e.g., sample size of young nestlings should be larger than that of grown nestlings, sample size of the smallest young should be smaller than that of others, and so on). Thus, to examine whether or not the effects of temperature on body mass depends on some factors, authors need to test the interaction between the grouping factor and temperature on body mass. Or, at the least, authors should explain why they did not test interaction terms. Finally, authors should present their main results in the main text rather than supplements (i.e., all statistical analyses should be shown in the main tables).

**Response.** Thank you for your thorough review and comments indicating where additional information or clarification is needed. We have responded to each of the issues you’ve raised below.

***Issue 1.*** The first issue is the lack of explanation. For example, I could not understand why authors compared between the smallest nestlings and others (instead of dividing nestlings into small-sized and large-sized groups). I guess, there must be a good reason to use this classification, but authors should carefully explain the logic.

***Response.*** We now include an explanation of why we chose to compare the smallest nestling to other nestlings (see below).

Pg 8, Lines 152-156: We choose to compare the smallest nestling to all other nestlings because, in North American barn swallows, incubation generally begins when the penultimate egg is laid [32]; As a result, it is common for one nestling to hatch ~one day later than its brood mates, and we expected the largest size disparity to be between this last hatched nestling and all others [33].

***Issue 2.*** Likewise, I could not understand why authors did not show mean temperature.

***Response.*** Thank you for bringing this to our attention. We now include the mean temperature during the nestling period in the “Background characteristics” section and S2 Table.

Pg 15, Lines 317-320: For the near-nest temperature measurements averaged across the nestling period, the mean ± SD was 23.99 ± 1.32 degrees C for mean temperature, 13.10 ± 1.80 degrees C for minimum temperature, 36.90 ± 2.96 degrees C for maximum temperature, and 9.26 ± 2.43 degrees C for temperature variability.

***Issue 3.*** And I could not understand why authors categorized high/med/low parental care instead of high/low parental care.

***Response.*** We now present the results of analyses where parental care is stratified into two levels (high / low). We additionally include the three-level analysis (high / med / low) as a sensitivity analysis to assess the robustness of our results to differences in the selected number of strata for parental feeding. The methods, results, and discussion have been updated to reflect the changes in analyses.

***Issue 4.*** Authors should also carefully explain correlations between variables. When correlation between variables is high, their relationships with dependent variables might not be independent (and thus they should be analyzed together, rather than separately).

***Response.*** We assessed the effects of minimum, maximum and variability of temperature separately based on our *a priori* hypothesis that these conditions would have separate effects on nestling growth. While we expect a correlation between minimum and maximum temperature, our question was not to assess the effect of each temperature extreme while holding constant the other temperature values. This approach also allows us to avoid issues with collinearity and overfitting our model. We have clarified the rationale for and limitations of modeling the three temperature variables separately in the “Limitations and Strengths” section (see below)

Pg 29-30, Lines 633-640: Second, we modeled three temperature variables (minimum, maximum, and variability) separately because they capture different aspects of the thermal environment, offering insight on the effects hot, cold, and variable temperatures on nestlings. Additionally, modeling these variables separately allowed us to avoid issues with collinearity and overfitting our model. However, given that we modeled the three temperature variables separately, we were unable to determine the relative strength of the effects of different temperature variables on nestlings or whether there are cumulative effects of different types of adverse temperature exposures (see discussion in [90,91]).

We chose to run separate models for our three questions (focused on developmental stage, relative nestling size, and parental feeding level) because we were interested in the overall effect of each of these variables, rather than the effect of each variable after adjusting for the other variables (which is what a model of all three variables together would tell us). Additionally, based on our study design, we did not expect correlations among these three variables (e.g., parental feeding is measured at the nest level, and we would not expect any correlation of parental feeding with relative nestling size because, excepting occasional mortality, all nests contain “min” and “other” nestlings).

***Issue 5.*** Second, authors should analyze interactions between variables. Statistical significance depends on sample size, and thus significant relationship in some groups but not in others does not always mean they differ in slope (e.g., sample size of young nestlings should be larger than that of grown nestlings, sample size of the smallest young should be smaller than that of others, and so on). Thus, to examine whether or not the effects of temperature on body mass depends on some factors, authors need to test the interaction between the grouping factor and temperature on body mass. Or, at the least, authors should explain why they did not test interaction terms.

***Response.*** For questions concerning effect modification, we now include models with an interaction term between temperature and the effect modifier, in addition to stratified analyses (see updated “Methods” text below).

Pg 13-14, Lines 282-292: To determine whether effect modification was present, we ran analyses stratified by multiple levels of relative nestling size or parental feeding. The results from these stratified models provide insight into the magnitude and directionality of the effect of temperature on nestling mass for different relative nestling sizes or levels of parental feeding. In addition, for each question, we ran a model with unstratified data including an interaction term between temperature and the effect modifier (relative nestling size or parental feeding). The estimate, confidence interval, and p-value for the interaction term were used to inform interpretation of stratified results. We present stratified results even in the absence of significant interaction terms, given that interaction terms can be insignificant when effect modification is present due factors such as low power, the scale of measurement selected, and presence of non-linear relationships [50–53].

***Issue 6.*** Finally, authors should present their main results in the main text rather than supplements (i.e., all statistical analyses should be shown in the main tables).

***Response.*** The main results are reported in-text and represented in the figures, making the model result tables redundant. We are concerned that including the model result tables in the main text may create confusion, as readers may wonder why we present the same results several times. For these reasons, we prefer to keep the main tables in Supplemental Material. However, if the Reviewer and Editor disagree, we can add the model results tables to the main text.

**Comment.** L33 the smallest nestling Please see above.

**Response.** Please see our response to the overall comment above (under “Issue 1”).

**Comment.** L36 early-life social environment I don’t understand what kinds of “social” environment, you mean here.

**Response.** We clarified the wording of this sentence.

Pg 2, Lines 34-37: These findings indicate the existence of fine-scale heterogeneity in which the effects of temperature on nestling development are sensitive to metabolic constraints and early-life social environment (e.g., size relative to siblings), providing insight into the factors that may ameliorate or exacerbate climate impacts on individual birds.

**Comment.** L55 key Why? Please explain.

**Response.** We clarified the wording of this sentence.

Pg 3, Lines 54-56: This knowledge would advance our understanding of heterogeneity in susceptibility of nestling birds to climate impacts and the factors that may ameliorate or exacerbate these impacts.

**Comment.** L87 the presence of helpers at the nest What this means? A little bit ambiguous, I think. Please rephrase (and perhaps a little bit in depth explanation is helpful, because authors regard this as a type of “parental” care).

**Response.** We clarified the wording of this sentence.

Pg 5, Lines 85-87: Similarly, studies of cooperatively breeding bird species suggest that the presence of helpers—additional, non-parent individuals that care for offspring (an example of alloparental care)—may mitigate negative impacts of weather on nestlings [18] (but see [29]).

**Comment.** L121 both parents in a social pair provide care Ambiguous expression (what “care” means, here?). Only females brood nestlings, I think.

**Response.** We clarified the wording of this sentence.

Pg 6, Lines 121-122: In this socially monogamous species, both parents in a social pair provision food until nestlings fledge (>18 days post hatch) [32].

**Comment.** L126 checked every three to five days Did you accurately estimate hatching asynchrony? I am a little bit skeptical, as five days old nestlings are already big.

**Response.** We believe our measure of hatch asynchrony to be accurate for several reasons. First, once nests were close to their estimated hatch date, we checked them every two days—we added text clarifying our nest monitoring methods (see below). Second, aging of nestlings was based on a previous study (cited in-text, see below) which provided detailed descriptions of developmental characteristics present in nestling barn swallows at each age. Taken together, our monitoring efforts and reliable estimation of newly hatched nestlings’ age allowed accurate assessment of hatch asynchrony.

Pg 6-7, Lines 126-131: Nests were checked every three to five days to track nestling phenology and fate, and when nests were close to their estimated hatch date (based on clutch initiation date), they were checked every two days. During the first check after nestlings hatched, we estimated their ages (hatch day = day zero) based on feather emergence and other reliable developmental characteristics, such as wetness (on hatch day) and ability to raise their head [35].

**Comment.** L132 temperature near the nest Ambiguous expression.

**Response.** We clarified the wording of this sentence.

Pg 7, Lines 133-134: We monitored temperature using loggers placed 10-28 cm from the nest from hatch through last nestling measures (~ day 12).

**Comment.** L151 wing length measures skeletal size Wing length is not “skeletal” size.

**Response.** We removed this phrase.

Pg 8, Lines 156-157: Our classification was based on right wing length, rather than mass, because wing length may not be as strongly influenced by short-term fluctuations as mass [37–39].

**Comment.** L191 low...med...high Please see above. I don’t know why authors used three-level categories. When there are multiple groups, readers should take into account multiple comparison issues (or at least explain whether or not P-value corrections are needed).

**Response.** We now include analysis of a two-level parental care variable. Please see our response to the overall comment above (under “Issue 3”).

We did not correct for multiple comparisons to account for multiple groups because the goal of this analysis was to assess consistency, or heterogeneity, of associations between temperature and growth among subgroups given the expectation that these effects have similar biological underpinnings. Accordingly, such correction would be overly stringent and has potential to inflate type 2 error.

**Comment.** L210 likely around six days Ambiguous information.

**Response.** We clarified the wording of this sentence

Pg 10, Lines 210-214: We chose these time periods based on previous research in swallows and other songbirds [14,44–46], which found that nestling swallows begin to develop the ability to thermoregulate independently around four to five days post-hatch, and the age of the effective homeothermy (defined here as ability to maintain relatively constant body temperature under natural conditions—in the nest with brood mates) is approximately six days of age.

**Comment.** L226 we balanced our inference What this means? Please explicitly write down.

**Response.** We removed ambiguous wording and explicitly defined how we report and interpret results.

Pg 12, Lines 239-244: We focused our reporting and interpretation of results on estimates of effect sizes and 95% confidence intervals, providing information on the strength and uncertainty of our effect estimates [47], as well as p-values interpreted using the language of evidence [48]. P-values less than or equal to 0.05 were interpreted as providing moderate to strong evidence for an effect, p-values between 0.05 and 0.10 as providing marginal evidence, and p-values greater than 0.10 as providing little to no evidence [48].

**Comment.** L238 we z-score Something wrong.

**Response.** We clarified the wording of this sentence.

Pg 12, Lines 249-251: For all models, we z-score standardized (subtracted the mean and divided by the standard deviation) numeric explanatory variables and covariates to aid in model fit and comparison of effect sizes.

**Comment.** L242 zero variance in some models This is not good reason to exclude site ID from other models.

**Response.** We have added additional justification of our choice to exclude site ID from our models. As explained below, site ID explained little or no variance across models, and its inclusion resulted in a singular fit for some models. As such, it was removed across models to avoid overfitting.

Pg 12, Lines 252-257: We assessed inclusion of a random effect for site ID to account for non-independence of measures taken at the same site, but this effect was removed to avoid model overfitting because it explained very little or zero variance across models, and its inclusion resulted in a singular fit for some models. Removal of site ID from models in which it explained a little variance did not substantially affect the direction, magnitude, or significance of effects.

**Comment.** L269 stratified Please see above. Authors should test interaction terms.

**Response.** Done. Please see our response to the overall comment above (under “Issue 5”).

**Comment.** L294 minimum...maximum How about mean temperature? The interpretation of minimum and maximum temperature depends on mean temperature (e.g., imagine max temperature in cold winter days and min temperature in hot summer day: readers do not know the average temperature during the study period in this region).

**Response.** We now include summary statistics for mean temperature – please see our response to the overall comment above (under “Issue 2”).

**Comment.** L305 proportion of time spent brooding This should be high at early nestling stage. I don’t know how authors test the relationship between brooding and feeding (e.g., how did authors control nestling stage using nonparametric methods?). Please explain a little in depth. And please also show sample size in each statistical analysis.

**Response.** We used a Spearman Rank Correlation to estimate the correlation between brooding and feeding across all observations because we expect that time spent brooding would limit time available for feeding. We did not control for nestling stage in this exploratory analysis because it is not possible to control for third variables in correlation analysis, and our goal was not to model the effect of nestling stage on the variables in question.

We now provide the sample size in-text for this correlation analysis. Sample sizes for all models are provided in the figure captions and supplemental tables.

**Comment.** Figs. 3–5 These are analyzed in the same statistics? If not, authors should explain how they statistically assess independent effect of each variable.

**Response.** We used separate models to address each of our questions – please see our response to the overall comment (under “Issue 4”) for further explanation of this choice.

**Comment.** L331 did not change substantively What “substantively” means here? Please explicitly explain.

**Response.** We clarified the wording of this sentence.

Pg 17, Lines 354-357: The magnitude, direction, and precision of estimates of interest did not change substantially when models were run using five days or seven days post-hatch, rather than six days post-hatch, as the cut-off for age for early versus late development or when influential outliers were excluded from analyses.

**Comment.** L397 we found some evidence for heterogenous effects What this means? Authors did not use interaction term.

**Response.** We now consider the interaction of temperature with relative nestling size and parental feeding level in our interpretations—please see our response to the overall comment above (under “Issue 5”).

We have also revised the wording of this sentence.

Pg 21, Lines 449-450: Additionally, we found some evidence that the effects of temperature on nestling mass differ across developmental stages and social environments.

**Comment.** L402 relative nestling size What authors tested is difference between the smallest and other nestlings.

**Response.** We clarified the wording of this sentence. Please also note that we define relative nestling size explicitly in the methods section: “Using day eight to nine measures (the earliest age at which we could begin tracking individuals), we created a categorical variable of relative nestling size, where the smallest nestling in each nest based on right-wing length was classified as ‘min’ (reference group) and the remaining nestlings were classified as ‘other’” (Pg 8, Lines 159-152).

Pg 21, Lines 453-455: In addition, we found marginal evidence that temperature has stronger negative effects on the smallest nestling in the nest than other nestlings.

**Comment.** L421 ...and social factors, such as habitat, diet, and nest type Which one is “social”?

**Response.** Thank you for pointing this out. We removed the word “social” from this sentence to reflect its focus on other ecological factors.

Pg 22, Lines 475-476: Ecological factors, such as habitat, diet, and nest type, may shape susceptibility to negative effects of temperature across species and populations [18,73].

**Comment.** L424 more strongly influenced Compared with what?

**Response.** We clarified the wording of this sentence

Pg 22, Lines 476-479: For example, insectivorous species may be more sensitive to extreme temperatures than granivorous species because insect availability is more strongly influenced by weather conditions than seed availability [74,75].

**Comment.** L431 ontogeny Why authors did not mention about brooding? Brooding should be more frequent in early than in late nestling stages.

**Response.** Thank you for pointing this out. We chose to focus on provisioning in this paper, rather than brooding. However, we tested the correlation of brooding and feeding behavior to inform our interpretation of results—please see our response to the comment on L305 above. Additionally, we have added consideration of the potential role of brooding in shaping temperature effects across development (please see our response to the next comment) and effect modification by parental care (see below) to our discussion.

Pg 28, Lines 603-614: While we and others have focused on feeding, feeding may co-vary with other parental care behaviors, such as brooding, that also influence nestlings’ responses to temperature exposure. If parental feeding and brooding positively covary, as some studies have reported [83,84], then any modification of temperature effects on nestling mass by parental care may be driven by active parental regulation of nest temperatures, in addition to or instead of access to higher energetic resources via food provisioning. However, in our study population (and other studies: [85,86]), feeding and brooding are negatively correlated, suggesting that there are trade-offs between these behaviors. Our inconclusive findings regarding effect modification by parental feeding, therefore, might be in part driven by such trade-offs—nests that receive high levels of parent feeding may receive lower levels of brooding compared to nests with low levels of feeding, making it difficult to disentangle what aspects of parental care modify the effects of temperature on nestling growth.

**Comment.** L453 limited ability to cool themselves...surface area I think, authors discussion is mainly based on logic (and previous studies) rather than direct observation. Nestlings swallows are brooded by mothers particularly when they are young (and thus low temperature should have particularly large effects when nestlings are young, because parents cannot provide foods while they are brooding). Nestlings open their mouth when they are hot (and thus not only body surface area but relative mouth size would affect thermoregulation). These behaviors should be taken into account when interpreting the main results.

**Response.** Thank you for raising these points. At your suggestion, we have added discussion of the potential roles of trade-offs between brooding and feeding and of mouth size in shaping temperature effects on nestlings across ontogeny.

Pg 23, Lines 489-502: Nestlings may be especially sensitive to cold temperatures before thermoregulatory independence because colder ambient temperatures result in colder body temperatures at times when the parents are away from the nest, and colder temperatures slow metabolic processes in young nestlings [76]. In older nestlings, improved thermoregulation allows for a more constant body temperature and metabolic rate increases when ambient temperatures are outside the thermal neutral zone [6]. Additionally, parents must brood young nestlings frequently, especially during cold temperatures, and they are generally unable to feed and brood nestlings simultaneously. This trade-off between brooding and feeding may lead to larger effects of cold temperatures on the growth of young than old nestlings. Third, due to their higher surface area to volume ratio and lack of feathers, young nestlings may dissipate heat more quickly than older nestlings, which may be a disadvantage under cold conditions [6,8,72]. Finally, changes in specific thermoregulatory behaviors (e.g., panting or shivering, [77,78]) and mouth surface area (which may affect evaporative cooling via panting) across ontogeny might also contribute to differences in temperature effects on young and old nestlings.

**Comment.** L461 relative size Please see above.

**Response.** We revised the wording of this subheading to more directly reflect our analyses.

Pg 24, Lines 520-521: Effects of temperature on nestling mass may differ for the smallest nestling compared to other brood mates

**Comment.** L487 hierarchy But, authors focus solely on the smallest nestlings (i.e., authors did not study size difference among “others”).

**Response.** We clarified the wording of this sentence.

Pg 26, Lines 553-556: However, in contrast to previous work, we found only weak evidence for effect modification by relative nestling size, which may reflect that the sibling size hierarchy (specifically, the difference in size between the smallest nestling and other nestlings) does not strongly affect nestling vulnerability to temperature in our study system.

**Comment.** L546 unable to track individual nestlings Then, authors cannot discuss about nestling hatching order (e.g., the last hatched nestlings; L474).

**Response.** Thank you for bringing this to our attention. We have checked to ensure that we do not discuss nestling hatch order when describing our findings. The example listed (previously L474) was a reference made to a separate study, which was able to determine hatch order, in our discussion.

**Comment.** L551 Ffith Typo

**Response.** Corrected.

**Comment.** Fig. 1 I don’t know whether authors could obtain these predictions before having their results (e.g., do two lines cross? If so, where they cross?). Their categorizations (e.g., early/late, smallest/others, low/med/high) needs explanations.

**Response.** We have addressed the comment about categorizations above – please see our response to the overall comment (under “Issue 1” and “Issue 3”).

Our predictions figure was meant to represent general patterns (e.g., stronger relationship of temperature with nestling mass in early development than late development), rather than specific details (e.g., exact intercept and slope). However, we based decisions about the figure, such as whether the lines crossed, on the design of our study. For instance, the lines do not cross in Question 2 because we expected the smallest nestling to have a lower mass than other nestlings, regardless of temperature (and what we were interested in is the slope of the line, not the intercept). Beyond these specifics, the predictions (e.g., the steepness of the slopes) are relative and meant simply to highlight expectations under scenarios of effect modification.

**Comment.** Figs. 3-5 Low-quality figures. Please use high-resolution figures (or vector files such as eps). Please show sample size for each category. Please do not use dot line (because it is often used for non-significant relationships and thus misleading).

**Response.** Thanks, we provided have provided high resolution figures. Additionally, we added sample sizes for each category to the figure captions. Finally, we no longer use the dot line in any figure.

**Reviewer #2**

**Comment.** This study investigates the effect of ambient temperature on nestling growth and how its effect depends on the age of the nestlings, brood size, and parental provisioning effort. Including ambient temperature data at each nest in the analysis is an innovative plus. Surprisingly few studies have considered the effect of temperature on different stages of nestling growth combined with parental provisioning effort and this study makes a valuable contribution by doing so.

My only concerns are already enumerated by the authors in their wonderful 'Limitations and strengths' section, so I will mention only that the issue of sample size and the fact that nestlings were handled before rather than after provisioning data were collected could be a potential problem. However, as the authors clearly identify these limitations, it will make it easy for readers to take them into account.

**Response.** Thank you for the positive comments, particularly your recognition of the ‘Limitations and Strengths’ section. We have addressed your line-by-line suggestions below.

**Comment.** line 43: hyphenate 'At risk'

**Response.** Done.

**Comment.** line 91: Add scientific name after barn swallow

**Response.** Done.

**Comment.** line 120: Add 'external' before morphological

**Response.** Done.

**Comment.** line 124: hyphenate 'first brood'

**Response.** Done.

**Comment.** line 145: clarify if two blood samples were collected at each of the two sample times or if one sample was collected at each of the two times

**Response.** We have clarified the wording of this sentence.

Pg 7, Lines 147-148: We also collected two small blood samples from each nestling at each of two time periods—on days 8-9 and 11-13—as part of a separate study [36].

**Comment.** line 513: separate 'maybe' into two words

**Response.** Done

**Comment.** line 551: replace 'Ffith' with Fifth.

**Response.** Done.

**Comment.** lines 552-555: Add statement that handling and drawing a blood sample may also have affected nestling begging behavior

**Response.** Done.

Pg 30, Lines 650-654: Finally, it is possible that parental care and / or nestling begging behavior was affected by collection of morphological and physiological data before our observations, though we attempted to minimize these effects by waiting until birds appeared to resume normal behavior before beginning parental care data collection.

**Comment.** References: format needs to be standardized

**Response.** Done.
