# Supplementary material for: The effects of temperature on nestling growth in a songbird depend on developmental constraints
Source: PLoS One. 2026 Apr 22;21(4):e0334815. doi: 10.1371/journal.pone.0334815 (PMC13102239; doi:10.1371/journal.pone.0334815)
Supplement: Attachment — Submitted filename: nestling_temperature_ms_pt_by_pt_revision2_final.docx [file pone.0334815.s013.docx]

**Point-by-point response to the editor’s and reviewers’ comments**

**Editor**

**Comment.** Overall, this ms is an interesting evaluation of the relationship between food provisioning and temperature on the growth of altricial birds. Having taken over this ms from the previous editor, I have read through the previous comments and responses. Both reviewers found merit in the work, with Reviewer 1 still having a number of comments to consider and reviewer 2 being satisfied. Most of Reviewer 1’s comments are straightforward to address and will improve flow of the ms. The issue of p-values is one that many scientists are on different views about and thus while you provided a citation supporting your approach, it was clear the reviewer does not agree with that view. In any case, as long as you have complete p-values shown, then I concur with your approach (though I would note that if you can provide a measure of fit or explanatory power that is helpful). Finally, I have a number of comments related to altricial bird growth and the environment that I listed below and will help to clarify your work. I look forward to seeing a revised ms.

**Response.** Thank you for your detailed and helpful comments on altricial bird growth and the environment. Additionally, thanks for your acknowledgement of the many different views surrounding p-values. We respond to each of your line-by-line comments below.

When working on revisions, it came to our attention that there was a mismatch in our statistical approach with that of a previous publication with the same set of authors. Specifically, the mismatch was in the unstratified model looking at the interactive effects of parental feeding BLUPs and temperature on nestling mass. In this MS, we had originally modeled the interaction using a continuous variable for parental feeding BLUPs. However, in the previous publication considering effect modification of the relationship of growth and physiological stress response by parental care (Laubach et al. 2025 *American Naturalist*), we instead modeled the interaction using a categorical variable for parental feeding level, where feeding BLUPs were divided into ‘high’ and ‘low’ categories. This categorical interaction matches more closely with the stratified analyses in both manuscripts, where we ran a separate model for each of the two parental feeding levels (high and low). As such, in this MS, we now model the interaction using a two-level categorical variable for parental feeding level. This choice ensures consistency with similar analyses in a previous publication and a better match of our unstratified and stratified analyses. Additionally, this approach is more consistent with our analysis for question two, in which we looked at the interactive effects of a categorical variable for relative nestling size and temperature on nestling mass.

**Comment.** L46. I would suggest describing species not strictly as passerine songbirds, but as altricial species. The topic you are interested in less specific taxonomic distinction and more on the developmental continuum.

**Response.** Done.

**Comment.** L50. How is temperature affecting food delivery? I would suggest separating out these ideas and explaining more. Precipitation or marked decrease in temperature are often what drives changes in food delivery.

**Response.** We now explain these ideas further in the Introduction paragraph focused on parental care.

Pg 5, Lines 97-104: Finally, adverse weather conditions may lead to reduced food provisioning. For instance, provisioning rates may decrease in hot or cold temperatures due to reduced availability of insect prey [11,32] and parents diverting more time and energy to their own thermoregulation [33,34]. Similarly, food availability and provisioning rates often decrease during periods of precipitation or high wind, especially for aerial insectivores [29,35–38]. Therefore, it is plausible that parental feeding influences nestling vulnerability to adverse conditions, including extreme temperatures, in altricial birds.

Additionally, we have added a discussion of the potential impacts of precipitation and other environmental conditions on the variables and relationships under study to the “Limitations and Strength” section.

Pg 28, Lines 600-602: Second, because our study focused on first broods in a single year, we were unable to investigate whether the patterns we observed are generalizable across broods (first versus later broods) or years.

Pg 29, Lines 625-629: Additionally, various environmental factors, such as precipitation and wind speed, may have affected the outcome (nestling mass) and relationships we investigated (e.g., see [28,38,97–99]). Although incorporating these additional environmental factors was beyond the scope of our analyses, future studies considering the combined effects of multiple environmental factors would be of great value.

In our study, we were interested in the extent to which varying levels of parental feeding summarized across development, differentially affected nestling vulnerability to extreme temperatures, rather than the effects of temperature on food provisioning itself. As such, we accounted for the potential effects of temperature on food delivery when creating the index of overall parental feeding levels and treated this variable as an effect modifier in our analyses.

Pg 10-11, Lines 211-226: We created an index of the level of parental feeding by summarizing feeding rates from both parents across all stages of nestling development. Specifically, we used a generalized linear mixed effects model, in which total feeding count by both parents measured at each developmental stage was the outcome and nest ID was a random intercept. To fit models, we used the ‘lme4’ package, version 1.1.35.5 [49] in R version 4.4.1 [50]. We compared models with several error distributions and link functions using diagnostic plots and tests from the package ‘DHARMa,’ version 0.4.6 [51]. We selected a negative binomial distribution and log link for our final model because this appropriately handled overdispersion in the count data. The model included an offset for total observation time and several covariates that could influence parental feeding behaviors: number of days since the first nestling hatched, number of nestlings in the nest, the median temperature near the nest during the observation period, and the duration of time between removing nestlings from the nest for morphometric and physiological measures and the start of the observation. From this model, we extracted the best linear unbiased predictors (BLUPs) (following [43]). Because parental feeding is an effect modifier in our models, we created a two-level categorical variable for stratified analyses by classifying BLUPs as ‘low’ (lowest half) or ‘high’ (highest half) parental feeding (S1 Fig).

**Comment.** L57. In altricial birds.

**Response.** Done.

**Comment.** L58. I would suggest adding in approximate days during which you consider young for a small passerine. For most altricial species this true early period (and for your species of study) is hatch to several days. But help the reader understand more about how you define time during the in nest growth period.

**Response.** We have removed the word “young” and instead focus on the timing of thermoregulatory development during the nestling period, as well as the potential effects of temperature on nestlings before or after endothermy develops.

Pg 3-4, Lines 60-72: The effects of temperature on altricial nestling development may differ depending on the timing of exposure during development [11–14]. During the first few days after hatch, nestlings are mostly featherless and lack the ability to thermoregulate independently [8,15]. Consequently, exposure to hot or cold ambient temperatures may drive body temperatures outside of the narrow range optimal for growth [5,6,11,13]. Endothermy typically develops over several days in the middle of the nestling period [6,15], with timing influenced by growth rate of the species, brood size, and a variety of other factors [5,15,16]. After endothermy develops, nestlings exposed to hot or cold temperatures may expend more energy on thermoregulation and/or enter a state of hyperthermia (in the case of extreme heat), leading to increased stress and decreased growth [6,17,18]. Because the thermoregulatory abilities of nestlings change over the course of the nestling period, we might expect the effects of temperature on nestling growth to vary across ontogeny. This possibility warrants further investigation, as most previous studies have focused on the nestling period as a whole (but see [11–14]).

**Comment.** L61. One item I’m a bit unclear here is are you meaning temperature increase or decrease or both? The reason that this is important as it often varies over the breeding season with early spring having bouts of cold snaps and later into summer having potential heat wave. Also, precipitation is often the other variable driver here of food provisioning rates and at least should be mentioned.

**Response.** We now specify that we are interested in the effects of exposure to both cold and hot temperatures.

Pg 3-4, Lines 60-69: The effects of temperature on altricial nestling development may differ depending on the timing of exposure during development [11–14]. During the first few days after hatch, nestlings are mostly featherless and lack the ability to thermoregulate independently [8,15]. Consequently, exposure to hot or cold ambient temperatures may drive body temperatures outside of the narrow range optimal for growth [5,6,11,13]. Endothermy typically develops over several days in the middle of the nestling period [6,15], with timing influenced by growth rate of the species, brood size, and a variety of other factors [5,15,16]. After endothermy develops, nestlings exposed to hot or cold temperatures may expend more energy on thermoregulation and/or enter a state of hyperthermia (in the case of extreme heat), leading to increased stress and decreased growth [6,17,18].

In addition, we provide information on potential effects of precipitation on food provisioning in the paragraph focused on parental care—please see our response to the comment on L50.

**Comment.** L63. Same comment as above. Give some approximation of percent of growth completed or number of days to reach this benchmark.

**Response.** Our understanding is that the timing of development of endothermy is variable within and across species. We now explain this with more nuance and provide a rough approximation of when during the nestling period endothermy usually develops. Please see our response to the comment on L58.

**Comment.** L69. Some, but most nestlings are born relatively close to together and typically have one that does not. How far apart in time does asynchrony matter here?

**Response.** Thank you for asking this question. We have revised this section to focus on hatching asynchrony where some nestlings hatch a day or two later than their brood mates, as this is a common pattern in altricial birds and was the focus of our study.

Pg 4, Lines 74-78: [19,20]. Altricial nestlings often hatch asynchronously, with some nestlings hatching a day or two later than their brood mates, as a result of incubation beginning before all eggs are laid [21]. Hatching asynchrony typically results in size asymmetry, with younger nestlings remaining smaller than their brood mates across development [21].

**Comment.** L81. Is it parental care or is it really just food provisioning or one of the parents sitting on the nest?

**Response.** We now include food provisioning as an example of parental care. Our study focuses on food provisioning which provides a direct resource benefit to nestlings. We do not focus on brooding or other parental care behaviors in this study, expect where they aid our inferences about feeding behavior.

Pg 5, Lines 87-92: Parental care, including food provisioning, is another aspect of the social environment that may shape nestling responses to adverse conditions [19,20,30]. Nestlings are entirely dependent on food provisioned by parents to provide energy for growth and thermoregulation. As such, nestlings that receive more parental care may be less susceptible to the negative effects of temperature and other adverse conditions because they have more energetic reserves for thermoregulation and growth.

**Comment.** L93. Figure acknowledgement should be moved to your hypotheses as it isn’t showing the questions you are testing, but the hypotheses.

**Response.** Based on another comment, we now provide the predictions in the main text and acknowledge the figure at that time.

**Comment.** L100. I would suggest that you move the predictions out of the figure and that they follow on their respective hypothesis. Also, for each of the predictions I would expect to see citations or deductive reasoning as to why you expect to see these relationships.

**Response.** We now provide the predictions in-text, following each hypothesis. Additionally, we provide reasoning for each set of hypotheses and predictions.

Pg 5-6, Lines 105-130: In wild barn swallows (Hirundo rustica erythrogaster), we asked three questions and explored related hypotheses about the effects of near nest temperature on nestling growth. First, we asked: Does the effect of temperature on nestling mass differ when the exposure is assessed during early versus late development? Given that temperature exposures during different stages of development differentially effect nestling metabolic rate and thermoregulatory strategies (e.g., [5,6,15]), we hypothesized that due to differences in metabolic costs, exposure to extreme and variable temperatures early in development would have a stronger effect on nestling mass than exposure later in development. We predicted that hot, cold, and variable temperatures from hatch to day five (early in development), before expected development of thermoregulatory abilities, would have stronger negative effects on nestling mass before fledging than temperatures from day six to twelve (late in development) (Fig 1).

Our second and third questions asked whether social experiences modify the effect temperature on nestling growth. For question two, we asked: Does the effect of temperature on nestling mass depend on whether a nestling is the smallest in the brood? For question three, we asked: Does the effect of temperature on nestling mass depend on the amount of feeding provided by parents? Differences in nestling size are associated with heterogeneity in access to resources [21–23] and can impact thermoregulatory ability [6,24]. In addition, previous studies report that parental care can buffer nestlings from effects of adverse conditions [19,31]. Therefore, we hypothesized that disadvantageous social environments (e.g., being the smallest in the brood, lower levels of parental feeding) would exacerbate the negative impacts of extreme and variable temperatures on nestling mass due to the nestlings’ reduced access to resources. We predicted that hot, cold, and variable temperatures would have stronger effects on nestling mass for the smallest nestling in the brood relative to other brood mates and that temperature exposure would have stronger effects on nestling mass at low than high levels of parental feeding (Fig 1). We set out to test these hypotheses in an exploratory nature, as a step toward understanding how the developmental and social conditions under study may shape temperature effects on nestlings.

**Comment.** L103. I would suggest just one figure that has the multiple panels be in main body of ms. No need for this sentence explaining that information.

**Response.** Done. Because these conceptual figures include the variables under study and are relevant to the specific linear mixed models we ran, we reference this new figure (Fig 3) in the “Statistical analysis” subsection of the methods.

**Comment.** L107. Cut this sentence from the figure legend.

**Response.** Done.

**Comment.** L114. One limitation of this work in relation to climate change is that you are studying only a single year. I would suggest that the key part of your work is really on intra-annual variability as that was all you are really going to be able to do.

**Response.** Thank you for bringing this up. We acknowledge that our dataset does not allow us to test explicit hypotheses about climate change per se. We have revised sentences in our introduction and discussion to focus more broadly on human-induced environmental changes that impact temperature extremes and variability.

Pg 3, Lines 56-59: This knowledge would help identify sensitive periods of development, when altricial nestlings are at greatest risk to extreme or variable temperatures, as well as the factors that may confer resilience to or exacerbate the impacts of human-induced environmental change.

Pg 30, Line 642-646: We provide a more complete picture of how different aspects of the thermal environment affect nestling growth, while broadening our understanding of how developmental and social factors may confer resilience to or ameliorate the impacts of human-induced environmental change on vulnerable individuals.

Additionally, given the limitations of our study, we have added the following sentence at the end of our introduction.

Pg 6, Lines 129-130: We set out to test these hypotheses in an exploratory nature, as a step toward understanding how the developmental and social conditions under study may shape temperature effects on nestlings.

Finally, we now note in the limitations section that our single-year study limits the generalizability of our results.

Pg 28, Lines 600-602: Second, because our study focused on first broods in a single year, we were unable to investigate whether the patterns we observed are generalizable across broods (first versus later broods) or years.

**Comment.** L117. Please give the mean and SD of clutch size.

**Response.** We now provide the mean and SD of clutch size for our study population in the Background Characteristics section.

Pg 17, Line 361: Clutch size was 4.31 ± 0.74 eggs.

**Comment.** L119. Since you have two negative statements here I would suggest using the words ‘neither’ and ‘nor.’

**Response.** Done.

**Comment.** L112. Give mean and SD for fledging in this species, ideally in your study location.

**Response.** Exact fledgling age is not tracked in our study location. However, we now provide an approximate mean and range for fledging age from other North American populations.

Pg 7, Lines 147-148: Fledging generally occurs around 21 days post-hatch (range 15 to 27 days) in North American populations [39].

**Comment.** L125. How was this timing of visitation decided? Five days is quite long for altricial nestling monitoring in relation to what you are studying.

**Response.** We included five days in the range for time between nest checks because this was the most conservative description of our methods—the time between nest checks never exceeded five days. Typically, we visited nests twice per week, with a given nest visited every three to four days. When nests were close to hatch, we visited more frequently. We have revised this section to clarify our nest check methods.

Pg 8, Lines 152-155: Nests were checked twice per week to track nestling phenology and fate—typically, we visited a given nest every three to four days, and the time between nest checks did not exceed five days. When nests were close to their estimated hatch date (based on clutch initiation date), they were checked every two days.

**Comment.** L133. Outside of the nest?

**Response.** We have revised this sentence to clarify that temperature loggers are placed outside of the nest.

Pg 8, Lines 160-161: We monitored temperature using loggers placed outside the nest, 10-28 cm from the nest edge, from hatch through last nestling measures (days 11-13).

**Comment.** L137. Cut this explanation sentence.

**Response.** Done.

**Comment.** L146. How were able to differentiate individuals before this point in time and isn’t that an important part of the study?

**Response.** Thank you for raising this point. We were unable to differentiate individuals before days 8-9 post-hatch, when they were banded with a USGS band. We tried several options for marking younger individuals, but we had various logistical difficulties, and we ultimately had to focus on other aspects of data collection due to the intensive nature of the project and the synchrony of hatches during the study year.

**Comment.** L147. How was blood collected and how much?

**Response.** We now provide additional information on blood sample collection.

Pg 9, Lines 172-175: We also collected two fasting blood samples from each nestling at each of two time periods—on days 8-9 and 11-13—as part of a separate study [43]. Each blood sample consisted of several drops of blood (<30 microliters), collected via venipuncture of the brachial vein [43].

**Comment.** L149. There are methods to tag/mark individuals younger than this age. Unclear why that wasn’t done.

**Response.** Please see our response to the comment on L146.

**Comment.** L159. Just curious why a lab experiment wasn’t attempted to actually control a lot of these pieces of information? The inexact aging here and lack of telling individuals apart is a big limitation.

**Response.** This is an interesting suggestion. Our study was conducted on population for which there was established annual field work, as part of a long-term study of barn swallows. For various reasons, barn swallows are difficult to rear in captivity, and we are not aware of any studies where barn swallows were reared in captivity.

However, we agree that there are limitations to our field study, and that a lab study could address some of these limitations in future work. In the Discussion, we now suggest that future studies should include lab experiments to combat these limitations.

Pg 28-29, Lines 610-613: Future studies could address many of these limitations by conducting experiments where the variables under consideration (e.g., temperature, brood hierarchy, food delivery) are controlled and manipulated directly by researchers.

**Comment.** L165. Is there precedent for monitoring after you’ve done the measurements rather than beforehand? I would have thought that beforehand would be better.

**Response.** Thank you for raising this point. We completed the measurements before observations because we needed fasting blood samples for another study. We acknowledge that instead completing observations before measurements would be ideal in future studies without this constraint. We now mention that we collected fasting blood samples specifically.

Pg 8, Lines 172-173: We also collected two fasting blood samples from each nestling at each of two time periods—on days 8-9 and 11-13—as part of a separate study [43].

We also discuss this limitation in the “Limitations and Strengths” section.

Pg 29, Lines 613-616: Finally, it is possible that parental care and / or nestling begging behavior was affected by collection of morphological and physiological data before our observations, though we attempted to minimize these effects by waiting until birds appeared to resume normal behavior before beginning parental care data collection.

**Comment.** L177. Given this situation why not just always use cameras?

**Response.** Due to hatch synchrony and access to a limited number of cameras, we had to divide people and cameras among our study nests.

**Comment.** L183. Need to indicate you are using r.

**Response.** Done.

**Comment.** L200. Ok, but already one struggle I’m having is how much of the data you are collecting is simply being put into categorical terms and not continuous. Growth and regulation is fundamentally a continuous process and much is being missed by a categorical approach.

**Response.** We coded all explanatory variables, including measures of temperature, and our outcome, nestling mass, as a continuous variables in all analyses. We categorized relative nestling size within a nest to compare the effect of temperature on future mass between the smallest versus largest. Preserving this relative within nest ranking, we based our categorization on natural cut point – the smallest nestling size. Similarly, we categorized parental care, which we also treated as an effect modifier variable to give us a better understanding of differences in and to improve interpretation of the effect estimates among levels of parental care.

**Comment.** L215. Did you measure days of inclement weather over the course of the study? Precipitation directly affects many aerial insectivores and thus feeding rate in your study and hence growth could easily be affected by it as much or more so than temperature.

**Response.** We did not measure days of inclement weather over the course of the study, though we acknowledge that this would be useful in future studies. We have now added discussion of the potential impacts of rain and wind to our “Limitations and Strengths” section.

Pg 29, Lines 625-629: Additionally, various environmental factors, such as precipitation and wind speed, may have affected the outcome (nestling mass) and relationships we investigated (e.g., see [28,38,97–99]). Although incorporating these additional environmental factors was beyond the scope of our analyses, future studies considering the combined effects of multiple environmental factors would be of great value.

Additionally, we did not conduct any parental care observations during rain or high wind, which we now indicate in the main text—please see our response to Reviewer 1’s comment on L167.

**Comment.** L241. Did you also calculate any measure of fit? Helpful to readers to see not just p-values and CI’s.

**Response.** We provide marginal and conditional R^2^ values for all stratified models in the supplementary tables. We have also clarified the wording of the methods section regarding measures of fit.

Pg 13, Lines 279-282: To assess goodness of fit, we estimated marginal R^2^ (variance explained by fixed effects alone) and conditional R^2^ (variance explained by full model, including fixed and random effects) for each model [58], using the package ‘MuMIn’, version 1.48.4 [59].

**Comment.** L264, L273, L298, L419, L570, L586. I would suggest changing the wording here as sensitivity analysis is a formal comparison in simulation modeling of parameter variation. You didn’t quite do that. Change wherever this issue occurs.

**Response.** Done.

**Comment.** L297, L331-3. Avoid stating a figure for reader to look up and instead explain what you mean and have the figure notation in parentheses.

**Response.** Done.

**Comment.** L318. Use degree symbol throughout.

**Response.** Done.

**Comment.** L320. Can cut as this is restating what you did. I would suggest cleaning up the Results section further as noted by the reviewer. Do not need to describe what you did or how you did it, just main findings and reporting of stats.

**Response.** Done.

**Comment.** L326. I would suggest that you end the stats section in the Methods by saying something like ‘All results are presented as means +/- SD, unless otherwise stated.’ This statement will cut some wording here and make it clear throughout the Results what is being reported.

**Response.** We have added this statement to the beginning of the “Background Characteristics” section, where it was most relevant, and cut down the wording of the section.

Pg 17, Lines 359-360: All results for background characteristics are presented as mean ± SD, unless otherwise stated.

**Comment.** L354, L440. Move this sentence up to previous paragraph and add it in. Paragraphs should be three sentences or more in length.

**Response.** Done.

**Comment.** L443. Cut this first paragraph and replace it with whether or not you found support for each hypothesis. Do not need to restate the reason for the research or sample size, etc.

**Response.** Done.

Pg 22-23, Lines 471-491: We found mixed support for our hypothesis that due to differences in metabolic costs, exposure to extreme and variable temperatures early in development would have a stronger effect on nestling mass than exposure later in development. Consistent with our hypothesis, we found that higher minimum temperatures in early but not late development--before but not after putative development of thermoregulatory independence at six days post-hatch—were associated with higher nestling mass (Fig 4a). However, contrary to our hypothesis, higher maximum temperatures and greater temperature variability in both early and late development—before and after putative development of thermoregulatory independence—were associated with lower nestling mass (Fig 4b-c).

Similarly, some of our results but not others supported our hypothesis that disadvantageous social environments exacerbate the negative impacts of extreme and variable temperatures on nestling mass due to the nestlings’ reduced access to resources. Consistent with our hypothesis, we found marginal evidence that minimum temperature, maximum temperature, and temperature variability have stronger effects on the mass of the smallest nestling than other brood mates (Fig 5), as indicated by marginally significant statistical interactions of temperature with relative nestling size. Additionally, in support of our hypothesis, minimum temperature had stronger effects on nestling mass in nests receiving low than high levels of parent feeding (Fig 6a), as evidenced by a significant statistical interaction of parental feeding level and minimum temperature. On the other hand, contrasting with our hypothesis, there was no evidence for quantitative differences in the effects of maximum temperature and temperature variability on nestling mass across levels of parental feeding (Fig 6c-d).

**Comment.** L483. And precipitation.

**Response.** We now consider the potential role of precipitation in our introduction and discussion of limitations (please see comments on L50, L61). Additionally, we note that rainfall affects insect availability earlier in this paragraph.

Pg 24, Lines 500-503: For example, insectivorous species may be more sensitive to extreme temperatures than granivorous species because insect availability is more strongly influenced by weather conditions, including rain, wind speed, and temperature, than seed availability [35,83,84].

**Comment.** L486, L503, L522. Move up to beginning of Discussion.

**Response.** Done. Please see our response to comment on L443.

**Comment.** L505, L513, L556. Need an object after the word ‘this’ so there is logical connection to previous sentence. Double check ms throughout for issue.

**Response.** Corrected and double checked throughout the MS.

**Comment.** L561. Reviewer 1 suggested a shorter Discussion and I concur. This section is one where you could cut down the length and just give succinct reasons, rather than some of the back and forth.

**Response.** Done.

**Comment.** L594. I would recommend that some of these studies be done in the lab where most of the factors can be controlled. It is possible to raise nestlings in the lab and vary temp, humidity, food delivery, etc. and monitor individuals.

**Response.** Thank you for this suggestion. We now suggest that future studies include lab-based approaches to address many of the limitations we discuss.

Pg 28-29, Lines 610-613: Future studies could address many of these limitations by conducting experiments where the variables under consideration (e.g., temperature, brood hierarchy, food delivery) are controlled and manipulated directly by researchers.

**Comment.** L631. For a field based growth study you have a pretty solid sample size. Yes, more is better, but I think you want to consider other reasons. Timing in the year when the work was done, effects of precipitation, if relationships vary year to year, etc. I think you are spending a lot of time on issues that are important, but could be mitigated by a more elegant lab based approach (or even better a lab-field combined study).

**Response.** Thank you for bringing this to our attention. We now consider additional factors in our discussion of limitations.

Pg 28, Lines 600-602: Second, because our study focused on first broods in a single year, we were unable to investigate whether the patterns we observed are generalizable across broods (first versus later broods) or years.

Pg 29, Lines 625-629: Additionally, various environmental factors, such as precipitation and wind speed, may have affected the outcome (nestling mass) and relationships we investigated (e.g., see [28,38,97–99]). Although incorporating these additional environmental factors was beyond the scope of our analyses, future studies considering the combined effects of multiple environmental factors would be of great value.

In addition, we suggest that future work includes lab-based approaches, as these address some of the limitations we discussed—please see our response to the comment on L594.

**Comment.** L671. Can cut this last section, no need to just restate findings. If there are implications of your work, that would be a different way to end the work.

**Response.** Done.

**Reviewer #1**

**Comment.** Overall: Thank you very much for your revision. The revised manuscript is better than the initial manuscript. However, I have some comments on the revised manuscript. First of all, authors should not regard non-significant results as statistical “support.” Only when authors had significant results (i.e., P < 0.05), authors can obtain statistical support. All descriptions using marginal (i.e., 0.05 < P < 0.10) as a support for the hypothesis should be deleted (or at the least, should not regarded as a support for the hypothesis). Authors should be objective throughout the text. Authors should also carefully write down their statistics (e.g., test statistics of interaction terms). Second, Authors’ discussion is too long. Rather than explaining each result one by one, authors should discuss overall patterns (based on objective statistical results). Please see below for each comment.

**Response.** Thank you for the second round of detailed comments. We have addressed each point below.

**Comment.** L21 avoid exposure Unclear (=ambiguous) expression. Please rephrase.

**Response.** Done.

Pg 2, Lines 20-21: Climate change can adversely impact animals, especially those that cannot independently thermoregulate or avoid exposure to temperature variation.

**Comment.** L26 mass Body mass would be better, I guess.

**Response.** Done.

**Comment.** L29 higher minimum temperature were associated with lower nestling mass Really? I think, “associated with higher nestling mass” would be a correct expression here. Please check.

**Response.** Thank you for catching this error. We have corrected the text.

Pg 2, Lines 29-31: Lower minimum temperatures were associated with lower nestling mass in early but not late development (before but not after putative development of thermoregulatory independence).

**Comment.** L31 marginal Please see above.

**Response.** In agreement with the editor, we believe scientists have many different opinions on cut-offs for P-values. We have chosen to discuss P-values in the language of evidence, following Muff et al. 2022 (*Trends in Ecology and Evolution*, doi:10.1016/j.tree.2021.10.009). Additionally, we present effect sizes and 95% confidence intervals, which provide more information on the direction, strength, and uncertainty of our effect estimates than do p-values, following Halsey 2019 (*Biology Letters,* doi:10.1098/rsbl.2019.0174). As discussed in these papers and references therein, the use of arbitrary p-values cut-offs and binary decision-making for hypothesis testing has several important issues, including focus on statistical significance over biological importance, irreproducibility of results, and misinterpretation of p-values. These issues can be addressed in part by taking a more nuanced and gradual approach to evidence. As such, our intent in the MS is not to be misleading, but instead to take a nuanced approach to reporting and interpreting our results that balances biological and statistical inference and, in addition, balances type 1 and type 2 error (noting our relatively small sample size). Finally, we have added a sentence in the introduction addressing the fact that our hypothesis testing is inherently exploratory and aims to encourage additional studies to build a body of evidence.

Pg 6, Lines, 129-130: We set out to test these hypotheses in an exploratory nature, as a step toward understanding how the developmental and social conditions under study may shape temperature effects on nestlings.

**Comment.** L46 development of adult phenotype Authors should explain why this is important (reproduction and later survivorship, right?).

**Response.** Done.

Pg 3, Lines 44-47: Exposure to excessively hot or cold temperatures is a form of developmental stress that negatively impacts not only the immediate survival of young animals in many species, but also the development of adult phenotypes that affect later survival and reproduction [3,4].

**Comment.** L58-L63 These expressions look redundant (i.e., authors used similar expressions multiple times). Please revise.

**Response.** We have revised this section.

Pg 3-4, Lines 61-69: During the first few days after hatch, nestlings are mostly featherless and lack the ability to thermoregulate independently [8,15]. Consequently, exposure to hot or cold ambient temperatures may drive body temperatures outside of the narrow range optimal for growth [5,6,11,13]. Endothermy typically develops over several days in the middle of the nestling period [6,15], with timing influenced by growth rate of the species, brood size, and a variety of other factors [5,15,16]. After endothermy develops, nestlings exposed to hot or cold temperatures may expend more energy on thermoregulation and/or enter a state of hyperthermia (in the case of extreme heat), leading to increased stress and decreased growth [6,17,18].

**Comment.** L65 most studies... as a whole, leaving unanswered...before versus after Unclear expression, I guess. Authors can change expression so that readers can easily contrast previous studies and the current study (i.e., To study the importance of developing thermoregulation, we don’t need to compare before vs after, e.g., including days after hatching as an independent variate, and thus the current expression would be misleading).

**Response.** We have revised this section.

Pg 4, Lines 69-72: Because the thermoregulatory abilities of nestlings change over the course of the nestling period, we might expect the effects of temperature on nestling growth to vary across ontogeny. This possibility warrants further investigation, as most previous studies have focused on the nestling period as a whole (but see [11–14]).

**Comment.** L83 burying beetles This is an insect species, and thus authors should carefully and briefly explain why this example can be used for understanding birds.

**Response.** We now include our reasoning for consideration of the burying beetle example.

Pg 5, Lines 92-94: Indeed, research on burying beetles (Nicrophorus vespilloides), an invertebrate that displays elaborate biparental care like many altricial bird species, indicates that higher levels of parental care can buffer young from effects of temperature variation [31].

**Comment.** L87 weather Do this “weather” means temperature? If so, please write it down. If not (e.g., rain), I am not sure why authors cite this article. Please explain.

**Response.** The study in question focused on periods of low rainfall, which we now state explicitly. We discuss this example because it demonstrates that parental care can buffer nestlings from the negative impacts of adverse conditions, which might include periods of low or high rainfall (depending on the species) and extreme temperatures, among other things. We have now broadened the paragraph to focus on how parental care may mitigate adverse conditions, including extreme temperatures as one example.

Pg 5, Lines 87-104: Parental care, including food provisioning, is another aspect of the social environment that may shape nestling responses to adverse conditions [19,20,30]. Nestlings are entirely dependent on food provisioned by parents to provide energy for growth and thermoregulation. As such, nestlings that receive more parental care may be less susceptible to the negative effects of temperature and other adverse conditions because they have more energetic reserves for thermoregulation and growth. Indeed, research on burying beetles (*Nicrophorus vespilloides*), an invertebrate that displays elaborate biparental care like many altricial bird species, indicates that higher levels of parental care can buffer young from effects of cold temperatures [31]. Similarly, studies of cooperatively breeding bird species suggest that the presence of helpers—additional, non-parent individuals that care for offspring (an example of alloparental care)—may mitigate negative impacts of periods of low rainfall on nestlings [19] (but see [30]). Finally, adverse weather conditions may lead to reduced food provisioning. For instance, provisioning rates may decrease in hot or cold temperatures due to reduced availability of insect prey [11,32] and parents diverting more time and energy to their own thermoregulation [33,34]. Similarly, food availability and provisioning rates often decrease during periods of precipitation or high wind, especially for aerial insectivores [29,35–38]. Therefore, it is plausible that parental feeding influences nestling vulnerability to adverse conditions, including extreme temperatures, in altricial birds.

**Comment.** L101 advantageous social environments (e.g., larger size Authors focus on “the smallest” (L96) and thus authors should focus on the smallest (rather than “others”) throughout the text.

**Response.** Thank you for making this point. We have revised our hypothesis as suggested.

Pg 6, Lines 123-125: Therefore, we hypothesized that disadvantageous social environments (e.g., being the smallest in the brood, lower levels of parental feeding) would exacerbate the negative impacts of extreme and variable temperatures on nestling mass due to the nestlings’ reduced access to resources.

**Comment.** L115 Boulder Please provide latitude, longitude, altitude.

**Response.** Done.

**Comment.** L138 and others ~six days Please use English expression rather than “~”.

**Response.** Done.

**Comment.** L154 ~one day Please see above.
**Response.** Done.

**Comment.** L167 Observation Please provide weather information. For example, authors did observation at rainy or windy day? Likewise, throughout the text, temperature and weather (e.g., rainy, windy) should be separated.

**Response.** Rain is rare in the morning in our study area, and we now explain in that text that we did not complete observations during rain or high wind.

Pg 10, Lines 195-197: We did not complete observations during rain or high wind conditions (observation mean wind speed = 0.15, range 0 to 4.02 m/s).

**Comment.** L180 generalized linear mixed effects model Please explain detailed information of the statistics (e.g., statistical program used). When using negative binomial distribution, authors should explain how they control for overdispersion (which is automatically corrected in some programs but not in others).

**Response.** Thank you for bringing this up. We now include information on the statistical program (R) and packages we used. Diagnostic plots and tests from the package ‘DHARMa’ indicated no issues with overdispersion when we used a negative binomial distribution.

Pg 10-11, Lines 214-218: To fit models, we used the ‘lme4’ package, version 1.1.35.5 [49] in R version 4.4.1 [50]. We compared models with several error distributions and link functions using diagnostic plots and tests from the package ‘DHARMa,’ version 0.4.6 [51]. We selected a negative binomial distribution and log link for our final model because this appropriately handled overdispersion in the count data.

**Comment.** L180 total observation time Please provide mean, min, and max observation time.

**Response.** We now provide the mean and range of observation lengths in the “Parental care observations” section.

Pg 9, Lines 195-197: Observations began between 5:45 and 8:00 am, and their length ranged from 50.00 to 62.58 minutes, with a mean of 59.70.

**Comment.** L185 number of days since the first nestling hatched Did authors assume that provisioning rate increase linearly with days? This might not be the case (please see L328). Please explain.

**Response.** Nestling age was treated as a continuous variable in this model. We acknowledge that the summary statistics in the “Background characteristics” section may suggest that provisioning rate does not increase linearly with age. However, because we ran a generalized linear model with a log link, the model assumes a linear relationship between the predictor variables and the *logarithm* of provisioning rate, not the raw values. When examining diagnostics plots for the model, we did not detect any patterns in the residuals suggesting violation of the linearity assumption.

**Comment.** L216 over the entire nestling period How this information is used? Please explain (because I assume that authors focused on temperature information before and after key date).

**Response.** We used this information in all our analysis for questions two and three. Only our analyses for question one used temperature calculated separately in early and late development. We now clarify the use of this information in our “Statistical Analyses” section.

P 16, Lines 339-344: For question two, modeled the effect of each temperature variable measured across the nestling period on nestling mass on days 11-13 stratified by nestling size, ‘min’ = the smallest nestling in the nest and ‘other’ = all other nestlings. For question three, we modeled the effect of temperature measured across the nestling period on nestling mass on days 11-13 stratified by parental feeding, which included two levels, ‘low’ = parental feeding below the 50^th^ percentile and ‘high’ = parental feeding above the 50^th^ percentile.

**Comment.** L242 equal to 0.05 P = 0.05 is marginal (i.e., P < 0.05 is significant).

**Response.** Please see our response to the comment on L31.

**Comment.** L271 mass on days 11-13 was the outcome Then, the effect of temperature on young nestlings would be diluted, which might affect the results.

**Response.** While you raise a good point, we were interested in focusing on the mass during our last measure before fledging. Nestling mass before fledging is relevant to later fitness, as offspring that leave the nest at smaller sizes are less likely to survive than their larger counterparts across many species (Ronget et al. 2018, *Biological Reviews,* doi:10.1111/brv.12329). As such, by examining the effects of temperatures during two different developmental periods on mass before fledging, we were able to gain insight into the potential impacts of temperature exposure during these periods on a fitness-relevant outcome.

**Comment.** L290 insignificant -> nonsignificant

**Response.** Done.

**Comment.** L318 degrees C Do these values include night-time temperature? Please explain (because female swallows sit on the nests without feeding during night, night-time and day-time temperature should be distinguished). Also, please use °C throughout the text (as in L336).

**Response.** We now use °C throughout the text.

We did not separate daytime and nighttime temperatures in our analysis. However, the maximum temperature is certainly during the day, when the parents are feeding nestlings. Additionally, the minimum temperatures in our study area usually occur around sunrise, at which time the birds have begun foraging and provisioning chicks. We acknowledge that there are various things the birds could be doing that further affect the relationships between temperature and growth, but these could occur during the day or night and are beyond the scope of our study.

**Comment.** L330 Spearman rank correlation This is wrong (as authors told that this analysis is conducted throughout the nestling period in their reply to my previous comment). Imagine that parents incubate more and needs less foods during early nestling period than during late nestling period. Then, without tradeoff between the two parental care behaviors (i.e., brooding and provisioning), there can be a negative relationship between them (because of nestling period as a confounding factor). Authors should control the confounding factor when focusing on the relationship between brooding and provisioning.

**Response.** Thank you for clarifying this point. We did not control for nestling stage in this exploratory analysis because it is not possible to control for third variables in correlation analysis. However, to address the issue you raised, we now estimate the correlation of feeding and brooding separately at each time point.

Pg 10, Lines 207-209: Using these data, we examined the correlation of feeding rate and the proportion of time spent brooding using Spearman rank correlation because both behaviors may influence nestling response to temperature.

Pg 18, Lines 374-377: Feeding rate and proportion of time spent brooding during the observation were negatively correlated when nestlings were 8-9 and 11-13 days old (days 8-9: n = 26, rho = -0.58, p = 0.002; days 11-13: n = 25, rho = -0.43, p = 0.03), but not when they were 3-4 days old (n = 25, rho = -0.25, p = 0.22).

Pg 27-28, Lines 588-590: However, in our study population and other studies: [92,93], feeding and brooding are negatively correlated (at 8-9 and 11-13, but not 3-4, days post-hatch in our study), suggesting that there are trade-offs between these behaviors.

**Comment.** L331 p = Why authors used lowercase p here (but used uppercase P in others)? Please revise.

**Response.** Corrected. We now use lowercase p throughout the MS.

**Comment.** L336 β = ... [95% CI: Please provide SE and test statistics (t) throughout the results section. I am also not sure why authors used bootstrap to estimate 95%CI. Because authors used t-values for estimating P-values, authors had SE values as well and can estimate 95% CI based on them.

**Response.** We have chosen to include the SE and test statistics in the Supporting Information, rather than in-text, because much of the information is redundant (e.g., the SE does not provide additional information beyond the 95% CI) and because when included in text this extra information broke the flow, making it harder for a reader to focus on the biologically relevant comparisons of interest.

We chose to use bootstrapping to estimate 95% confidence intervals because some of our sample sizes are relatively small, and bootstrapped CIs do not rely on the same asymptotic assumptions as the default profile-based CIs estimated using lme4.

**Comment.** L344 Please explain why authors could not test interactions here (so that readers can easily understand the reason).

**Response.** We did not test interactions for question one because we were interested in whether there was a sensitive period where temperature exposure at different times during development affects the outcome, nestling mass. We did not model the interaction between early and late nestling temperature because we were not trying to test whether the effect of temperature at one developmental time period depends on temperature at another time period.

**Comment.** L355 substantially Please rephrase “substantially” with objective statistical terminology. For example, significant and non-significant relationship remains unchanged? What is “substantial” differs between researchers (and thus can be somewhat subjective) and thus authors should avoid using these kinds of words. I also did not understand what “precision” here means.

**Response.** We have revised this section and explicitly defined precision.

Pg 19, Lines 399-402: The magnitude, direction, and precision (width of CI), and significance of estimates of interest were similar when models were run using five days or seven days post-hatch, rather than six days post-hatch, as the cut-off for age for early versus late development and when influential outliers were excluded from analyses.

**Comment.** L364 and others interaction... β = Authors should test the interaction term (e.g., by comparing likelihoods of models with and without interaction terms), rather than testing the difference in slope in the model with interaction term.

**Response.** Our hypotheses were about effect modification; therefore, we were not trying to assess goodness of fit via likelihood ratio tests in the models with or without the interaction terms. Moreover, likelihood ratio tests are often not recommended for testing the significance of fixed effects in mixed effects models because they are based on asymptotic assumptions and may have inflated type 1 error, especially for small sample sizes. Additionally, likelihood ratio tests cannot be performed when model parameters are estimated using restricted maximum likelihood, as is the default for mixed effects models run using the R package ‘lme4’. As such, t- and F-tests with Satterthwaite or Kenward-Roger degrees of freedom estimation are considered more appropriate for these models. For additional discussion of these issues, please see Bolker et al. 2009 (*Trends in Ecology and Evolution,* doi:10.1016/j.tree.2008.10.008) and references therein.

**Comment.** L365 marginal support Please see my comment above.

**Response.** Please see our response to the comment on L31.

**Comment.** L373 β = 0.37 Why authors did not use unit here? Please describe results in the same manner throughout the text.

**Response.** Corrected.

**Comment.** L373, 374, 395 and others was (...) Please revise. Please do not use parenthesis like that.

**Response.** We have revised these sentences.

**Comment.** L385 only “Smallest” or “Other” nestling points are present for some nests. Then, authors should exclude these nests, as they experience changing social environment (i.e., not comparable with other nests in which social environments were unchanged).

**Response.** This situation only occurred in one nest. Due to our limited sample size, we chose to include as many nests in our analysis as possible. As such, we prefer not to exclude this nest.

**Comment.** L428 Please provide statistics for interaction terms here.

**Response.** We have moved the results from the three-level parental provisioning models, including statistics for the interaction terms, to the Supporting Information because the purpose of these additional analyses is to highlight consistency in the results from models where there are slight differences in how we parameterized parental provisioning. We feel that moving these results removes redundancy and helps streamline the main text, while still being transparent about slight differences in specific estimates and p-values that reflect expected statistical noise stemming from differences in power between the different models.

**Comment.** L433 were associated with ... P = 0.07 P = 0.07 is not significant.

**Response.** Please see our response to your comment on L31. Following the approach described therein, we have double-checked that p-values between 0.05 and 0.10 are reported as marginal evidence.

**Comment.** L441 substantially Please see above.

**Response.** Please see our response to your comment on L355. We have made the same revisions here.

**Comment.** L442 Discussion Too long.

**Response.** Thank you for pointing this out. We have shortened and restructured the discussion.

**Comment.** L456 was inconclusive, but... may be suggestive of This expression looks somewhat subjective (because authors stress positive results alone). Authors should also explain multiple testing. It is not surprising that some of many tests had “significant” results by chance.

**Response.** We have deleted the sentence in question.

We did not correct for multiple comparisons to account for multiple groups because the goal of this analysis was to assess consistency, or heterogeneity, of associations between temperature and growth among subgroups given the expectation that these effects have similar biological underpinnings. Accordingly, such correction would be overly stringent and has potential to inflate type 2 error.

**Comment.** L465-473 This part looks redundant. Please revise.

**Response.** Done.

Pg 23, Lines 494-497: Our collective set of results suggests that exposure to lower minimum temperatures, higher maximum temperatures, and greater temperature variability were associated with lower nestling mass, consistent with literature reporting that exposure to cold [11,73,74], hot [75–77], and variable temperatures [78] may all lead to reduced nestling growth.

**Comment.** L478 weather I guess, authors used weather as a synonym to temperature, but they are not identical. Authors should distinguish weather (e.g., sunny, cloudy, and so on) and temperature, even if they are correlated.

**Response.** We now specify that “weather” in this case includes rain, wind, and temperature.

Pg 24, Lines 500-503: For example, insectivorous species may be more sensitive to extreme temperatures than granivorous species because insect availability is more strongly influenced by weather conditions, including rain, wind speed, and temperature, than seed availability [35,83,84].

**Comment.** L508 Early in development...Late in development This contrast looks strange because authors found similar pattern regardless of developmental period (L503). Authors should explicitly explain why they found similar pattern regardless of the contrasting thermoregulatory issues between early and late developmental periods. It should also be noted that each nest might experience similar temperature condition between early and late nestling periods (and thus the observed pattern would be cumulative effects rather than independent effects of temperature in the each nestling period).

**Response.** Thank you for making these points. We have revised this section as suggested.

Pg 24-25, Lines 526-546: Our finding that higher maximum temperatures and greater temperature variability in both early and late development were associated with lower nestling mass suggests that nestlings may be sensitive to heat and environmental instability regardless of thermoregulatory status. However, the mechanisms underlying this temperature sensitivity may shift across ontogeny. Early in development, nestlings may be susceptible to negative impacts of extreme heat because they may gain heat more quickly than older nestlings due to their high surface area to volume ratio and lack of feathers [6,8,81]. Late in development, after development of thermoregulatory independence, nestlings may be sensitive to hot temperatures because evaporative cooling is energetically costly and may quickly lead to dehydration [77,88]. In addition, nestlings may have limited ability to cool themselves [88], which may be exacerbated in older nestlings by reduced heat dissipation due to higher surface area to volume ratio, feather development, and crowding in the nest (though reduced heat transfer may be an advantage when ambient temperatures exceed body temperature). Finally, the similar patterns we observed across time periods may have been influenced by positive correlations between temperatures in early and late development for maximum temperature and temperature variability. These correlations suggest that nests may experience similar temperature conditions in early and late development, and our findings may reflect the consistent effects of these environmental conditions across development on mass, rather than the separate effects of conditions during each developmental stage. Taken together, these developmental-stage-specific mechanisms and similar temperature conditions across development may explain why hot and variable temperatures negatively impacted nestlings in both early and late development.

**Comment.** L522 marginal Please see above.

**Response.** Please see our response to your comment on L31.

**Comment.** L531-L541 This paragraph resembles with L465-473. Please do not explain each result one by one. Authors should explain overall pattern in the discussion.

**Response.** We have revised this paragraph as suggested.

Pg 26, Lines 549-560: Our findings suggest that, although nestlings seem to experience negative effects of extreme and variable temperatures regardless of their relative size, the magnitude of these effects may be greater for the smallest nestling in the nest than other nestlings, as indicated by the marginally significant temperature by size interaction terms. There are several possible explanations for why the magnitude of temperature effects on nestlings may differ according to their relative size within a nest. First, animals with a smaller body size are more vulnerable to extreme temperatures because they are less able to regulate their body temperature—they dissipate heat more quickly when ambient temperature is below body temperature, and they absorb heat more quickly when ambient temperature exceeds body temperature [6,24]. This explanation involving thermoregulatory constraint aligns with studies of other species finding that extreme temperatures have stronger effects on the survival [27] and growth [22,29] of the smallest or last hatched nestling than other brood mates.

**Comment.** L557 relatively small Please explain why authors think that the difference between 26.8 and 32.4 was small (for me, >15% difference is large).

**Response.** We have deleted the sentence in question.

**Comment.** L576 p-values were above the cut-off for marginal evidence Please note that cut-off point is P = 0.05.

**Response.** We have removed this sentence. Please also see our response to your comment on L31.

**Comment.** L587 may indicate effect modification, even in the absence of a significant interaction term This is not good. Authors should objectively judge their results based on statistics and clear logic (please do not subjectively pick up your favorite results while ignoring others).

**Response.** We have removed this section of the discussion. Please see our response to the comment on L31 for explanation of our approach to reporting and interpreting results.

**Comment.** L594 non-linear relationships Then, instead of using categorization, authors can directly use estimate of provisioning rate across nestling period.

**Response.** We used categorization of explanatory variables to give us a better understanding of differences among levels of these variables. Modeling provisioning rate across the nestling period as a continuous variable would not allay concerns about non-linear relationships, as we would still be using a linear model. In fact, coding provisioning as a categorical variable releases us from the linearity assumption to some degree, as it means that we are looking at differences among group means, rather than the slope of a line. Regardless, we have deleted this section of the discussion.

**Comment.** L609 negatively corelated, suggesting that there are trade-offs Please see above.

**Response.** Please see our response to the comment on L330.

**Comment.** L611 nests that receive high levels of parent feeding may receive lower levels of brooding This is not shown (statistics across developmental period does not clarify patterns across nests in the same developmental period).

**Response.** Please see our response to the comment on L330.

**Comment.** L615-L628 This paragraph is hard to understand. Please rewrite.

**Response.** In the interest of shortening the discussion, we have deleted this paragraph and incorporated important information elsewhere in the MS.

**Comment.** L648 degrees of hatch asynchrony might be related to nest temperature during incubation This is beyond the scope of the current study. Also, authors should use “hatching asynchrony” instead of “hatch asynchrony.”

**Response.** We agree that this is out of scope of the current study, and as such, we discuss this point in the limitations section. We now use “hatching asynchrony” instead of “hatch asynchrony” as requested.

**Comment.** L673 differed among three temperature variables This is not surprising.

**Response.** We have removed this sentence.

**Comment.** Please use vector files.

**Response.** We have provided high-resolution TIFF files which meet the journal’s requirements and were approved by the software provided by the journal to check figure specifications.

**Comment.** Fig. 2 I like illustrations in this figure, but illustration of fledging swallows should be revised (because fledglings do not have long tails). It would be better to add the exact days when they fledge.

**Response.** Done.

**Comment.** Fig. 3-5 Please do not use broken lines.

**Response.** We chose to use lines of different styles to increase accessibility of our figures for people with visual disabilities. Additionally, the use of multiple styles makes the differences between lines more apparent when printed in black and white. In the last round of revisions, we removed dotted lines, as requested. However, we prefer to keep the dashed lines for the reasons stated above, unless the editor disagrees.

**Comment.** Supplementary Tables I am not sure why authors would like to put these tables in supplementary materials. Although authors respond to my previous comment that tables are redundant to in-text statistics, but then, authors should use tables rather than in-text statistics. Readers can easily access to statistics to tables (rather than in-text statistics). And authors should use “standard” table styles used by other articles.

**Response.** We prefer to represent the main results with estimates listed in-text and corresponding figures. This way the results section of the paper follows the organization of our hypotheses, while providing both a visual representation and exact values for the effect size and uncertainty of estimates of interest. We provide additional details about our modeled results in the Supporting Information. Finally, the tables follow a standard style, in so far as we are aware.

**Comment.** Statistics Authors did not report correlation between variables. This is not good. For example, how temperature variables in the early nestling period are correlated with those in the late nestling period? If they are tightly correlated (e.g., nests in variable temperature environment in the early nestling period experience variable temperature in the late nestling period, too), it is not surprising that similar pattern can be found in early and late nestling periods. Of course, authors know the pattern, and thus they should report correlations among variables (and explain why authors did not statistically control for correlations among variables). I am also not sure why authors did not include these variables in the same model.

**Response.** We modeled the effects of temperature in early and late development separately because we were interested in the overall effects of temperature at each developmental stage on mass, rather than the effect of each variable after adjusting for the other variables. Additionally, modeling these variables separately allowed us to avoid issues with collinearity and overfitting our model. As requested, we now provide the correlations between temperatures in early and late development.

Pg 15, Lines 311-312: To aid our inferences, we examined the correlation of each of our temperature variables between early and late development using Spearman rank correlation.

Pg 19, Lines 402-406: For minimum temperature, temperatures in early development were not significantly correlated with temperatures in late development (n = 31, rho = 0.26, p = 0.15). On the other hand, there was a significant positive correlation between temperatures in early and late development for maximum temperature (n = 31, rho = 0.57, p = 0.0007) and temperature variability (n = 31, rho = 0.81, p < 0.0001).

Additionally, we discuss these correlations when interpreting our results.

Pg 25, Lines 540-543: These correlations suggest that nests may experience similar temperature conditions in early and late development, and our findings may reflect the consistent effects of these environmental conditions across development on mass, rather than the separate effects of conditions during each developmental stage.
